# Hazard Assessment of Polymeric Nanobiomaterials for Drug Delivery: What Can We Learn From Literature So Far

**DOI:** 10.3389/fbioe.2019.00261

**Published:** 2019-10-23

**Authors:** Sandra Jesus, Mélanie Schmutz, Claudia Som, Gerrit Borchard, Peter Wick, Olga Borges

**Affiliations:** ^1^Center for Neuroscience and Cell Biology, University of Coimbra, Coimbra, Portugal; ^2^Laboratory for Technology and Society, Empa Swiss Laboratories for Materials Science and Technology, St. Gallen, Switzerland; ^3^School of Pharmaceutical Sciences, University of Geneva, Geneva, Switzerland; ^4^Laboratory for Particles-Biology Interactions, Empa Swiss Laboratories for Materials Science and Technology, St. Gallen, Switzerland; ^5^Faculty of Pharmacy, University of Coimbra, Coimbra, Portugal

**Keywords:** hazard assessment, exposure assessment, *in vivo* toxicity, oxidative stress, genotoxicity, toxicity on reproduction, hemocompatibility, polymeric nanobiomaterials

## Abstract

The physicochemical properties of nanobiomaterials, such as their small size and high surface area ratio, make them attractive, novel drug-carriers, with increased cellular interaction and increased permeation through several biological barriers. However, these same properties hinder any extrapolation of knowledge from the toxicity of their raw material. Though, as suggested by the Safe-by-Design (SbD) concept, the hazard assessment should be the starting point for the formulation development. This may enable us to select the most promising candidates of polymeric nanobiomaterials for safe drug-delivery in an early phase of innovation. Nowadays the majority of reports on polymeric nanomaterials are focused in optimizing the nanocarrier features, such as size, physical stability and drug loading efficacy, and in performing preliminary cytocompatibility testing and proving effectiveness of the drug loaded formulation, using the most diverse cell lines. Toxicological studies exploring the biological effects of the polymeric nanomaterials, particularly regarding immune system interaction are often disregarded. The objective of this review is to illustrate what is known about the biological effects of polymeric nanomaterials and to see if trends in toxicity and general links between physicochemical properties of nanobiomaterials and their effects may be derived. For that, data on chitosan, polylactic acid (PLA), polyhydroxyalkanoate (PHA), poly(lactic-co-glycolic acid) (PLGA) and policaprolactone (PCL) nanomaterials will be evaluated regarding acute and repeated dose toxicity, inflammation, oxidative stress, genotoxicity, toxicity on reproduction and hemocompatibility. We further intend to identify the analytical and biological tests described in the literature used to assess polymeric nanomaterials toxicity, to evaluate and interpret the available results and to expose the obstacles and challenges related to the nanomaterial testing. At the present time, considering all the information collected, the hazard assessment and thus also the SbD of polymeric nanomaterials is still dependent on a case-by-case evaluation. The identified obstacles prevent the identification of toxicity trends and the generation of an assertive toxicity database. In the future, *in vitro* and *in vivo* harmonized toxicity studies using unloaded polymeric nanomaterials, extensively characterized regarding their intrinsic and extrinsic properties should allow to generate such database. Such a database would enable us to apply the SbD approach more efficiently.

## Introduction

Over the last decades, several nanomaterials (NMs) have been developed and studied as promisor drug delivery vehicles and medical devices, including magnetic, metallic, ceramic and polymeric nanomaterials. At present, there is fragile consensus regarding the “nano” definition among different regulatory organizations. In detail, considering medical regulatory authorities, such as the European Medicines Agency (EMA) or the United States Food and Drug Administration (FDA) some considerations can be made. In a reflection paper about nanotechnology-based medicinal products for human use published in 2006, EMA defined nanotechnology as “the production and application of structures, devices and systems by controlling the shape and size of materials at nanometer scale,” considering that “the nanometer scale ranges from the atomic level at around 0.2 nm (2 Å) up to around 100 nm” (European Medicines Agency, 2006). On its turn, FDA guidance for considering whether an FDA-regulated product involves the application of nanotechnology (Food Drug Aministration, 2014) refers that it should be considered “the evaluation of materials or end products engineered to exhibit properties or phenomena attributable to dimensions up to 1,000 nm, as a means to screen materials for further examination and to determine whether these materials exhibit properties or phenomena attributable to their dimension(s) and associated with the application of nanotechnology.” Therefore, for the context of academic research and to the context of this review the following definition of nanomaterial applies: materials in the size range of 1 nm to 1,000 nm and a function or mode of action based on its nanotechnological properties. In addition, by “nanobiomaterial” we considered NMs intended to interact with biological systems. The application of nanobiomaterials in the medicine field present several advantages as they can (Moritz and Geszke-Moritz, 2015; Banik et al., 2016):

- Transport higher drug payloads
- Enable targeted drug delivery
- Increase the bioavailability of poorly water-soluble drugs
- Promote controlled drug delivery
- Increase the stability of drugs in biological fluids
- Increase drug circulation time in the body
- Confer drugs protection from biological fluids
- Permeate through various biological barriers
- Enable surface modifications to increase interaction with biological targets.

Considering polymeric NMs in particular, they can be assembled in different pharmaceutical nanosystems, such as nanoparticles (NPs), dendrimers, polymeric micelles and drug conjugates (Bhatia, 2016). On its turn, polymeric NPs comprise both vesicular systems (nanocapsules) and matrix systems (nanospheres) (Bhatia, 2016). The polymeric nature of these NMs provides additional advantages that are worth exploring, such as enhanced biocompatibility, biodegradability and low immunogenicity (Egusquiaguirre et al., 2016; Rana and Sharma, 2019).

All considered, most of these advantages are frequently attributed to their distinctive size which contributes to their high surface area to mass ratio, and is also responsible for the different toxicokinetic fate of the NMs (Landsiedel et al., 2012; Boyes et al., 2017). Indeed, small sizes facilitate cell uptake, penetration through endothelial and epithelial cells, interaction with tissues and accumulation in the liver, kidney and spleen (Khan and Shanker, 2015). The increased cellular interaction can have a modulatory effect on the immune system, triggering inflammation, increased susceptibility to infectious diseases, or even to autoimmune diseases or cancer (Kononenko et al., 2015).

The unique physicochemical properties of the NMs restricts the extrapolation of toxicological data from raw materials, and makes it necessary to have specific toxicological studies adequate to the nanoscale (Ge et al., 2011). Moreover, there is a need for specific and optimized methods for NMs toxicity evaluation, since interactions between NMs and current toxicity testing protocols can lead to false positive or false negative results (Khan and Shanker, 2015; Kononenko et al., 2015).

Understanding the toxicokinetics of NMs and their modulation of the immunological system is necessary to implement their Safe-by-Design based on the literature. This is an up-to-date subject, currently widely discussed among the scientific community, but most commonly for metallic NM (Gatto and Bardi, 2018; Kanwal et al., 2019).

Therefore, the objective of this review is to summarize what is known about the toxic effects of polymeric NMs, with special focus on polymeric NPs that could be correlated to human health risks. We intend to identify the analytical and biological tests described in the literature used to assess NMs toxicity and to evaluate and interpret the available results. Furthermore, we intend to understand the obstacles and challenges related to the nanomaterial testing that are still preventing a harmonized regulation on polymeric NMs for drug delivery and biomedical applications.

We started this review by discussing the pillars of human health risk assessment: exposure assessment and hazard assessment. Next, in order to analyze the state of the art about the toxic effects of polymeric NMs, peer reviewed original research articles from the last 10 years were analyzed and discussed, addressing the following endpoints: (1) *in vivo* toxicity (acute and repeated-dose), (2) oxidative stress, (3) inflammation, (4) genotoxicity, (5) toxicity on reproduction and (6) hemolysis. Importantly, articles were carefully examined regarding minimal characterization parameters, such as chemical composition, particle size, surface charge and endotoxin contamination (when relevant).

## Pillars for Human Health Risk Assessment

To perform human health risk assessment of any material is necessary to integrate the exposure assessment with hazard assessment. The first intends to determine routes of exposure and estimate exposure dosages (dose, duration and frequency) while the second intends to characterize the possible hazards (toxic effects) of polymeric NMs when in contact with the human body.

### Exposure Assessment

**Figure 1:**
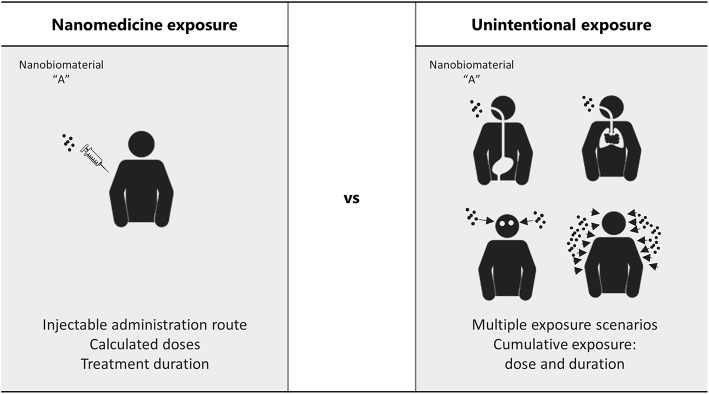
Hypothetical scenarios of exposure: comparison between the administration route and doses foreseen in medical applications and the exposure routes and cumulative doses difficult to predict in unintentional exposures, such as occupational scenarios.

Human exposure to polymeric NMs should be considered in the context of intentional nanomedicine applications, and in the context of occupational exposures of workers during the manufacturing processes, testing methods, distribution and handling/administration of polymeric NMs. Moreover, it cannot be disregarded situations where misuse and overuse are easily attained (Sayes et al., 2016). While in nanomedicine exposure scenarios, the administration route, the dose and duration of the exposure are well-defined, occupational exposure can happen through multiple and non-expected routes (Figure 1) and result in potentially cumulative levels of exposure and organ accumulation, whose impact in human health might be very different from the one predicted (Sayes et al., 2016). In fact, working with NMs involves challenges different from when working with bulk size materials, since they have increased ability to enter the human body, particularly through the respiratory airways, and to be translocated to the bloodstream and different organs (Yah et al., 2012). The lack of testing methods to detect and quantify the unintentional absorbed cumulative doses of these materials in the organism is currently, one of the main difficulties for designing predictive toxicological assays for occupational exposures. Therefore, exposure modeling arises as one alternative to allow occupational risk assessment. In the context of the FP7 NanoReg project a number of risk assessment tools for manufactured NMs, such as the CB NanoTool, the Nanosafer, and the Stoffenmanager-Nano have been examined and a new two-box nano specific exposure model (I-Nano) has been implemented (Jiménez et al., 2016). However, the need to rely on detailed input data (rate of particulate release from the source as well as the particle size distribution) which is not always available and its only application to inhalable exposures are some of the limitations present (Jiménez et al., 2016).

In the main, the NM routes of administration and exposure include respiratory, oral, ocular, dermal, and parenteral (injectable and implantable), each route presenting its own biodistribution pattern, resulting in different effects on human health. Indeed, the same composition, size and surface charge of the polymeric NM, might produce a different effect only by changing the exposure route (Sharma et al., 2016; Boyes et al., 2017). Importantly, it cannot be disregarded that the characteristics of the individual exposed, such as its age and health status, might also influence the NMs effect (Boyes et al., 2017). Table 1 below summarizes the most common administration/exposure routes and the most important characteristics of NMs related to each one.

**Table 1:** Common routes of administration/exposure: important considerations relating nanomaterials characteristics and the various routes of exposure (Agrawal et al., 2014; Blanco et al., 2015; Date et al., 2016; Palmer and DeLouise, 2016; Boyes et al., 2017).

**Route of exposure**	**Considerations on the exposure route**	**Nanomaterials characteristics and its relation with the exposure route**	
Respiratory	- The most common route of exposure in the workplace - Nanomaterials inhaled for drug delivery must overcome bronchial mucociliary clearance - Inhaled nanomaterials may translocate to various regions of the brain, without crossing the blood–brain barrier - Inhaled nanomaterials can cross the alveoli–blood barrier, reaching the systemic-circulation portion of the cardiovascular system, without gastric passage or a first-pass metabolism	Size	Particles of about 20 nm have the highest proportional deposition rate in the alveolar region Particles smaller than 55 nm will penetrate the alveoli more efficiently than particles of 200 nm or greater
		Charge	Positively charged nanomaterials will exhibit greater interaction with the mucus' negative charge, thus avoiding fast mucociliary clearance
		Others	Inhalation flow-rate influences which region of the respiratory tract nanomaterials will reach The mucoadhesive properties of nanomaterials may increase their residence time in nasal mucosa, increasing drug absorption
Oral	- The first choice, non-invasive route - Inhaled nanomaterials cleared by the mucociliary system may be ingested - Ingested nanomaterials can reach and interact with different organs of the GI tract, such as the esophagus, stomach, small and large intestine and colon - Ingested nanoparticles can translocate into the systemic-circulation portion of the cardiovascular system, but to do so they must resist a wide range of pH environments and enzymatic degradation until they reach the small intestine - The absorption of ingested nanomaterials can be hindered by the poor permeability of the intestinal epithelium - Before reaching systemic circulation, ingested nanomaterials and cargo drugs will undergo a first-pass metabolism in the liver	Size	Particles with a diameter of <50 nm are known to cross epithelial barriers via paracellular passage, whereas larger particles are endocytosed by intestinal enterocytes (<500 nm) or taken up by M cells in Peyer's patches (<5 mm)
		Charge	Positively charged nanomaterials may exhibit greater interaction with intestinal mucus, therefore improving nanoparticle retention, but also decreasing nanoparticle absorption Neutrally charged nanomaterials diffuse more efficiently through the mucus layers
		Others	Surface coating nanomaterials with enteric polymers improves their resistance in the gastrointestinal (GI) tract Hydrophilicity and poor chemical or enzymatic stability in the GI tract diminish intestinal absorption
Injectable	- Most commonly used routes for injectables include intravenous, intramuscular, subcutaneous and intradermal administration - Injectables are the first choice for active pharmaceutical ingredients with narrow therapeutic indices, poor bioavailability or administration to unconscious patients - Intravenously injected nanoparticles are distributed throughout the circulatory system, reaching different organs - Intradermal injection leads to uptake by the lymphatic system - Intramuscularly injected particles are taken up via the neuronal and lymphatic systems - Intravenously injected nanoparticles are rapidly cleared by the kidneys and liver, or via the reticuloendothelial system (res)	Size	Smaller nanomaterials are mostly absorbed into capillaries, whereas larger nanomaterials are drained by the lymphatic system
		Charge	Nanomaterials with positively charged surfaces exhibit greater interactions with blood components and are therefore more rapidly cleared by the mononuclear phagocyte system Nanomaterials with neutral and negatively charged surfaces have longer circulation half-lives
		Others	Nanomaterial surface hydrophobicity increases interaction with blood components and therefore increases nanomaterial clearance via the mononuclear phagocyte system Nanomaterial surfaces coated with hydrophilic polymers or surfactants exhibit decreased clearance by opsonisation
Dermal	- Mostly used for the topical delivery of molecules intended to act locally (sunscreens, antifungals, anti-inflammatory or keratolytic agents, etc.) - Accumulation in hair follicles can increase the penetration of nanomaterials and cargo drugs - Damaged skin is more permeable to larger nanomaterials - Small, lipophilic molecules can penetrate easily into the skin and eventually reach the bloodstream or the lymphatic system	Size	Nanomaterials <20 nm may penetrate or permeate intact skin Nanomaterials <45 nm may penetrate damaged skin Nanomaterials >45 nm may translocate or be stored in skin appendages (i.e., air follicles)
		Charge	Cationic nanoparticles have an affinity for the negatively charged skin pores (which can limit their subsequent diffusion)
		Others	Physicochemical methods, such as the application of low-frequency ultrasound or surfactants (i.e., sodium lauryl sulfate), are used to disturb the skin barrier and promote nanomaterial absorption

### Hazard Assessment

The NMs toxic effects might occur in the administration site or they can result from the nano-sized materials ability to cross biological barriers (mucosal barriers, air-blood barrier, blood-brain barrier, placenta barrier) reaching cells and tissues that are generally protected from bulk size materials (Buzea et al., 2007; Ai et al., 2011). This improved penetration of nanoparticles may increase the toxicity, but at the same time be advantageous in order to improve current therapies.

The uncertainties about using NMs for drug delivery and other biomedical applications result mainly from particle size reduction which is linked to increased reactivity and augmented toxicity (Ai et al., 2011). Nonetheless, several other properties can contribute to the effects of these nano-sized delivery systems, such as chemical composition, hydrophobicity/hydrophilicity, surface charge or shape. In the literature, there is a significant amount of data relating physicochemical features of NMs with cellular interaction, biodistribution, cytotoxicity and immune system activation, as reviewed elsewhere (Fröhlich, 2012; Ma et al., 2013; Salatin et al., 2015; Hoshyar et al., 2016; Jindal, 2017; Zhang et al., 2017). Nevertheless, general conclusions indicating toxicity trends for a specific nanoparticle physicochemical property, are limited to cautious hypotheses, only verified in particular scenarios (i.e., depending on the administration route, dose metrics, etc.). A review published in 2014 by Gatoo et al. (2014) discusses the correlation between the physicochemical properties of NMs and its toxicity. Briefly, smaller particles are often correlated with a higher toxicity, due to their increasing ability to cross biological barriers and reach different organs without being recognized by the reticuloendothelial system (RES) (Gatoo et al., 2014). Other characteristics, such as the non-spherical shape or the positive surface charge are also believed to contribute to an increased toxicity of NMs (Gatoo et al., 2014). Importantly, most of these conclusions are based on studies using inorganic NMs. Since chemical composition is one of the variables affecting the NMs toxicity, different behaviors can derive from the polymer composition and therefore, extensive extrapolations among all classes of NMs should be avoided. Moreover, most toxicity trends consider one characteristic at a time, but it is important to consider a holistic approach of the NM: all physicochemical characteristics are interconnected and together will influence its toxicological profile.

The key aspect to test polymeric NM for human toxic effects is the simulation of realistic human exposures. Those scenarios are difficult to simulate mainly due to: (1) the difficulty on transposing accurately human effective doses to *in vitro* settings; and (2) the difficulty to have complex *in vitro* systems, based on human cells or primary cell lines, that mimic the physiological complexity of the human body and its interaction with the materials (Sharma et al., 2016). Actually, most of the results of the application of *in vitro* studies to polymeric NMs might not reflect the realistic exposures, since the tests are performed at much higher concentrations than those that can be achieved in *in vivo* experiments (Landsiedel et al., 2017). Moreover, *in vitro* testing commonly use mass-based exposure metrics, which is believed to be a limiting factor, as particle number, surface areas and the formed agglomerates in suspension greatly influence the effective concentration delivered to cells (Hinderliter et al., 2010; DeLoid et al., 2014).

The intrinsic and distinctive characteristics inherent to the nanoscale dimension, might interfere with reagents and detection methods of *in vitro* assays recommended for bulk materials (Dobrovolskaia et al., 2009). For instance, NMs may bind to the marker enzyme lactate dehydrogenase (LDH) or they may interact with dyes and dye products, such as neutral red and the tetrazolium salt (MTT) (Landsiedel et al., 2017). On the other hand, polymeric NMs also go through modifications when in contact with biological matrices, such as: bio-corona formation, aggregation/agglomeration, dissolution, generation of new nano-sized particles (as a result of ionic salvation or degradation of surface coatings) (Sharma et al., 2016). These transformations of the NM can interfere with its toxicological effect, and most of the times are not considered during *in vitro* testing. Lastly, the selection of relevant positive and negative nano-sized controls is most of the times ignored, mainly because there is no clear knowledge-base on the toxicity (and especially immunotoxicity) of the different NMs (Dobrovolskaia and McNeil, 2013).

It is widely accepted that *in vitro* assays based on cell lines are an inexpensive and direct method to evaluate nanoparticle related toxicity in target tissues. However, results significantly depend on the chosen cell line (commonly immortalized cancer cells), incubation time, cell culture media or cell culture supplementation (Lorscheidt and Lamprecht, 2016). For instance, cell culture media supplementation with serum is highly likely to induce a protein corona in the surface of positively charged nanoparticles, changing its size and zeta potential, and therefore modifying the nanoparticle-cell interaction and uptake, and ultimately its biological effect (Khang et al., 2014; Lorscheidt and Lamprecht, 2016).

Overall, despite the great effort in developing high-throughput *in vitro* assays, there is still much variables to accurately mimic real exposure scenarios, and the results are often in disagreement with those of animal studies (DeLoid et al., 2014). Even so, nanotechnology laboratories are still searching for the best *in vitro* assays to replace *in vivo* testing and predict real exposure scenarios. This issue has been extensively discussed by Dobrovolskaia and McNeil (2013).

The urge to replace *in vivo* testing of toxicity, is motivated by the high costs and relatively low throughput of the assays, the inter-species variability particularly on the structure and function of the immune system, the low sensitivity of standard *in vivo* toxicity tests toward mild immunomodulation reactions, and most importantly, the ethical concerns about animal use (Dobrovolskaia and McNeil, 2013).

Altogether, it is widely accepted that efficient and cost-effective toxicological testing is required (DeLoid et al., 2014). For that reason, international organizations including OECD and ISO have developed official papers with considering the NMs properties and their influence on testing methods (Sharma et al., 2016; Dusinska et al., 2017).

In 2006, the OECD started a nanosafety programme overseen a Working Party on Manufactured Nanomaterials (WPMN), which aims to promote international cooperation on the human health and environmental safety of manufactured NMs, and involves the safety testing and risk assessment of manufactured NMs. Over the years they have published numerous reports and some test guidelines which are published in the OECD Series on the Safety of Manufactured Nanomaterials to provide up-to-date information on the OECD activities in this area (OECD^1^).

In 2005, the Technical Committee ISO/TC 229 was created. It aims at the standardization in the field of nanotechnologies. The specific tasks of this committee include developing standards for terminology and nomenclature, metrology and instrumentation, test methodologies, modeling and simulations, and science-based health, safety, and environmental practices (Behzadi et al., 2014). Over the years, the committee has published several standards, from which we can highlight the recent ISO/TS 19006:2016 [Nanotechnologies-5-(and 6)-Chloromethyl-2′,7′-Dichloro-dihydrofluorescein diacetate (CM-H2DCF-DA) assay for evaluating nanoparticle-induced intracellular reactive oxygen species (ROS) production in RAW 264.7 macrophage cell line] and the ISO 19007:2018 (Nanotechnologies–*in vitro* MTS assay for measuring the cytotoxic effect of nanoparticles), discussed below (Bazile et al., 1995; Behzadi et al., 2017). In addition to the specific standards generated by this committee, in 2017, the part 22—Guidance on nanomaterials, was implemented in ISO 10993 (Biological evaluation of medical devices) (Barratt, 2000). Although this technical report represents the current technical knowledge related to NMs for medical devices it does not contain detailed testing protocols.

An important contribution to this field is being given by the US National Cancer Institute Nanotechnology Characterization Laboratory, whose main objective is to facilitate the development and translation of nanoscale particles and devices for clinical applications. In fact, they have described several protocols for *in vitro* characterization as well as for *in vivo*, and for the physicochemical characterization of NMs (Assay Cascade Protocols—https://ncl.cancer.gov/resources/assay-cascade-protocols). In parallel, the European Nanomedicine Characterization Laboratory (EUNCL) is also developing standard operating procedures (SOPs) to allow the physical, chemical, *in vitro* and *in vivo* testing of nanobiomaterials (http://www.euncl.eu/).

## Hazard Characterization of Polymeric Nanomaterials—Literature Review

NMs toxicity should be evaluated by *in vivo* and *in vitro* assays considering its effect in the host physiological and immunological integrity (Yildirimer et al., 2011). Most of *in vitro* assays available for testing a NM toxicological effects are focused on the molecular mechanisms underlying toxicity (i.e., oxidative stress generation and inflammation), while *in vivo* assays, particularly acute and repeated dose toxicity assays assess the effects on vital organ functions [i.e., biomarkers of liver function, such as aspartate aminotransferase (AST) and alanine aminotransferase (ALT)].

Table 2 summarizes the studies collected from the literature of the last 10 years, assessing the toxicity of polymeric NMs for the endpoints studied. The polymers considered for analysis were chitosan, polylactic acid (PLA), polyhydroxyalkanoate (PHA), poly(lactic-co-glycolic acid) (PLGA) and policaprolactone (PCL). From the table systematization we can highlight three main issues: (1) chitosan based NPs are the most studied polymeric NMs followed by PLGA based NPs; (2) the different colors illustrating the generation or absence of effect for each endpoint according to the different studies, reflects the inconsistency in the results found for the same type of NM; (3) No data on PHA based NMs is available regarding those endpoints. The inconsistent results must be carefully analyzed because in fact they may be complementary results, as the NM characteristics, their concentrations, the cellular and animal models used and even the experimental methodology are significantly different among authors. Therefore, in the next sub-chapters each endpoint and respective studies will be discussed in detail in an attempt to scrutiny possible toxicity trends for polymeric NMs. To note, over the following discussion, the effect of some other polymers, such as alginate, polyethylene glycol (PEG), pluronic and polyvinyl alcohol (PVA) are addressed as they are often used as surface coatings and blends in chitosan, PLGA, PLA and PCL based nanomaterials.

**Table 2:**
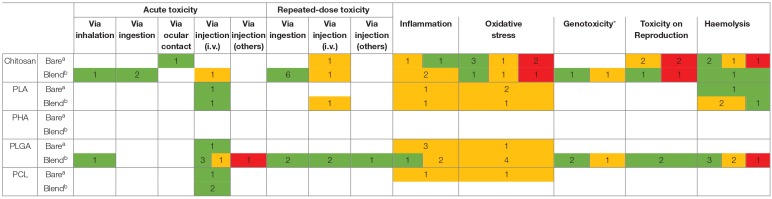
Systematization of the toxicity results described in the literature for chitosan, PLA, PHA, PLGA, and PCL nanomaterials.

*The number in each cell represents the number of studies supporting each conclusion according to the following color scheme: red indicates studies where all the concentrations tested induced an effect; orange indicates studies where at least one concentration tested induced an effect; green indicates studies that revealed no toxicity for any of the concentrations tested; (blank) no data available. Further details on each study are described in Tables 3–8*.

*^a^“Bare” polymer nanomaterials produced using crosslinkers or surfactants only, and which were not loaded with drugs, genes or proteins*.

*^b^“Blend” polymeric nanomaterials, functionalized/chemically modified polymers or particles loaded with drugs, genes, or proteins)*.

^*^Genotoxicity includes Mutagenicity and Carcinogenicity.

### *In vivo* Toxicity Studies

To study the toxicity of the NMs and to identify possible risks to the human health, researchers perform *in vivo* tests in animals (most time non-primates) to evaluate acute and repeated-dose (subacute, sub-chronic or chronic) toxicity. These studies, although highly valuable to understand the adsorption, distribution, metabolism and excretion (ADME) of the NMs as well as the immune system interactions, should be limited to a minimum according to the 3Rs strategy (replacement, reduction and refinement) (Oostingh et al., 2011; Dusinska et al., 2017). To note, in 2018, OECD guidelines for the testing of chemicals were adapted to accommodate the testing of NMs (OECD, 2018b,c).

As illustrated in Table 3, the available research articles testing *in vivo* the toxicity of NMs are characterized by a great variability between the rodent's species (or other animals, such as carps) used in the assays, the number of days (for the repeated-dose toxicity studies) and even for the endpoints that are analyzed. Some of the most reported endpoints are the clinical appearance of the animal, clinical signs of infection, hematological parameters, serum hemoglobin levels and albumin/globulin ratio, organ weights, and enhanced histopathology evaluation different organs (Dusinska et al., 2017).

**Table 3:** Review of original articles assessing *in vivo* the toxicity of polymeric nanoparticles.

**Nanomaterial**	**Polymer characterization**	**Nanomaterial characterization**	**Testing method**	**Model**	**Administration route**	**Dose/concentration range**	**Results**	**References**
Chitosan NPs	Chitosan hydrochloride salt (Protasan CL 110)	289 nm + 36 Mv (phosphate buffer)	*In vivo* exposure (acute toxicity)	New Zealand rabbits	Ocular	30 μL of the 0.5 mg/mL CSNP formulation in the right eye every 30 min for 6 h	No signs of discomfort in rabbits eyes 24 h after the administration No histopathological changes in the eye compared to control	de Salamanca A et al., 2006
Insulin (ins) loaded alginate/chitosan (Alg/chi) NPs	Depolymerized chitosan (65 and 25 kDa, and 86% DD^a^) Alginate (M/G^b^ content 64.5/35.5%)	3:1:1^c^ 104 nm, + 4 mV 3:2:1^c^ 157 nm, + 10 mV 3:3:1^c^ 216 nm, + 16 mV	*In vivo* exposure (acute toxicity)	Swiss albino mice	Oral	150 mg/kg b.w. (ratio alg:chi:ins 3:1:1)	No mortality No change in biochemical or histopathological parameters No liver or renal toxicity	Mukhopadhyay et al., 2015
Eudragit^®^ S100/alginate-enclosed chitosan-calcium phosphate-loaded lactoferrin nanocapsules	na	240 nm −2.6 mV	*In vivo* exposure (acute toxicity: 24 h)	*Artemia salina* (brine shrimp)	Oral (diluted in the water)	20–5,000 μg/mL	No lethality	Leng et al., 2018
Pluronic coated PLGA NPs	75:25 Resomer^®^ RG756 and Pluronic F68	240 nm −35 mV	*In vivo* exposure (acute toxicity)	Balb/cJ mice	Intratracheal (nebulization)	250 μg/50 μL in 5% glucose	Coated PLGA NPs did not induce an inflammatory response in mice, with no alterations of cellular population, protein quantity or expression of cytokines in BAL	Aragao-Santiago et al., 2015
PVA coared PLGA NPs	75:25 Resomer^®^ RG756 and PVA (87–89% hydrolyzed, 30–70 kDa)	220 nm −4 mV	*In vivo* exposure (acute toxicity)	Balb/cJ mice	Intratracheal (nebulization)	250 μg/50 μL in 5% glucose	Coated PLGA NPs did not induce an inflammatory response in mice, with no alterations of cellular population, protein quantity or expression of cytokines in BAL	Aragao-Santiago et al., 2015
Chitosan coated PLGA NPs	75:25 Resomer^®^ RG756 and Protasan^®^ UP CL113,75–90% deacetylation, 50–150 kDa	200 nm + 18 mV	*In vivo* exposure (acute toxicity)	Balb/cJ mice	Intratracheal (nebulization)	250 μg/50 μL in 5% glucose	Coated PLGA NPs did not induce an inflammatory response in mice, with no alterations of cellular population, protein quantity or expression of cytokines in BAL	Aragao-Santiago et al., 2015
Dissulfiram loaded PLGA nanoparticles, coated with PEG and functionalized with folate	PLGA (RG 504 H, acid terminated, lactide:glycolide 50:50, Mw: 38,000) and PEG-bis-amine (Mn: 10,000)	204 nm −5.24 mV	*In vivo* exposure (acute toxicity)	BALB/C mice	Intravenous	Equivalent to 120 and 60 mg/kg b.w. of dissulfiram	No lethality, no hematological parameters changes *(2,000 mg/kg of loaded NPs ~100 mg/kg equivalent of disulfiram)*	Fasehee et al., 2016
Dissulfiram loaded PLGA nanoparticles, coated with PEG and functionalized with folate	PLGA (RG 504 H, acid terminated, lactide:glycolide 50:50, Mw: 38,000) and PEG-bis-amine (Mn: 10,000)	204 nm −5.24 mV	*In vivo* exposure (acute toxicity)	BALB/C mice	Intraperitoneal	Equivalent to 2,000 and 225 mg/kg b.w. of dissulfiram	No lethality, hematological parameters altered *(2,000 mg/kg of loaded NPs ~100 mg/kg equivalent of disulfiram)*	Fasehee et al., 2016
Poly(ε-caprolactone)-poly(ethylene glycol)-poly(ε-caprolactone) (PCEC) nanoparticles	PCEC copolymer with a molecular weight of 17,500 (1H NMR spectrum)	40 nm	*In vivo* exposure (acute toxicity)	Sprague-Dawley rats	Intravenous	2.4 g/kg (divided in 2 administration within 12 h)	No clinical symptoms 14-days post-injection No histopathological findings after animal's sacrifice	Huang et al., 2010
Paclitaxel loaded PLA NPs	Inherent viscosity 0.55–0.75 dL/g and average molecular weight 75,000–1,20,000	150–175 nm, and zeta potentials lower than −15 mV	*In vivo* exposure (acute toxicity)	Wistar rats	Intravenous	10 mg/kg b.w.of paclitaxel	No induction of histopathological alterations (number, arrangement and architecture of cells) of the heart, lungs, liver, spleen, kidney, and brain Blank nanoparticles (unspecified dose) did not cause any toxicity as well	VasanthaKumar et al., 2014
Paclitaxel loaded PLGA NPs	Lactide:glycolide 50/50 and average molecular weight 5000–1,5000	150–175 nm < -15 mV	*In vivo* exposure (acute toxicity)	Wistar rats	Intravenous	10 mg/kg b.w.of paclitaxel	No induction of histopathological alterations (number, arrangement and architecture of cells) of the heart, lungs, liver, spleen, kidney, and brain Blank nanoparticles (unspecified dose) did not cause any toxicity as well	VasanthaKumar et al., 2014
Paclitaxel loaded PCL NPs	Average molecular weight 14,000 and average molecular number 10,000	150–175 nm, and zeta potentials lower than −15 mV	*In vivo* exposure (acute toxicity)	Wistar rats	Intravenous	10 mg/kg b.w.of paclitaxel	No induction of histopathological alterations (number, arrangement and architecture of cells) of the heart, lungs, liver, spleen, kidney, and brain Blank nanoparticles (unspecified dose) did not cause any toxicity as well	VasanthaKumar et al., 2014
Danorubicin loaded polyethylene glycol-poly L-lysine-poly lactic-co-glycolic acid (PEG-PLL-PLGA) NPs	na	229 nm −20 mV	*In vivo* exposure (Acute toxicity)	Kunming mice	Intravenous	40, 30, 22, 17, and 13 mg/kg b.w.of Danunorubicin (DNR) loaded in the particles	LD_50_: 464.4 mg/kg b.w.(23.22 mg/kg b.w.of DNR) 95% confidence interval: 399–542 mg/kg b.w.(20–27 mg/kg b.w.OF DNR) No significant pathological changes of organizational structure and cell morphology	Guo et al., 2015
Danorubicin loaded polyethylene glycol-poly L-lysine-poly lactic-co-glycolic acid (PEG-PLL-PLGA) NPs	na	229 nm −20 mV	*In vivo* exposure (Acute toxicity)	Kunming mice	Intravenous	200 mg/kg b.w.of DNR loaded in the particles	No lethality No physical signs of toxicity No changes in hepatic or renal markers	Guo et al., 2015
Amphotericin loaded PEG-PLGA nanoparticles	Copolymer produced with 6,000 Da PLGA (lactic to glycolic acid molar ratio of 1:1) and 15% PEG	25 nm	*In vivo* exposure (acute toxicity)	Albino Sprague-Dawley rats	Intravenous	Equivalent to 1 mg/kg of amphotericin and blank NPs	No nephrotoxicity (evaluated by renal injury biomarkers BUN and PCr) Although described no results presented for blank nanoparticles group	Radwan et al., 2017a
Angiopoietin-2 (Ang2) small interfering (si)RNA plasmid chitosan magnetic nanoparticles (CMNPs)	Chitosan polysaccharides (Mw^d^ 1,38,0000, 90% DD)	na^e^	*In vivo* exposure (acute toxicity)	Kunming mice	Intravenous	92, 153, 255, 424, and 707 mg/kg b.w.	All doses: no mortality, no changes in b.w. Higher doses: short-term staggering, reduced activities and accelerated breathing, as well as transient reduction of eating, lung uneven dark red coloring and particles aggregated inside the lungs *Based on the conversion method of equivalent dose co efficient, the non-toxic dose in humans should be <222 mg/kg per day for 14 day, overall a total of 3117 mg/kg, which is significantly higher compared with the quantity required clinically*	Shan et al., 2017
Tween 80 modified chitosan nanoparticles (TmCS-NPs)	Chitosan (100 kDa, 85% DD)	251 nm +26.5 mV	*In vivo* exposure (7 days)	Sprague-Dawley rats	Intravenous	3, 10, and 30 mg/kg b.w.	Body weight of rats remarkably decreased dose-dependently Dose-dependent neuron apoptosis and slight inflammatory response in the frontal cortex, and downregulation of GFAP expression in the cerebellum Study aim: neurotoxicity	Yuan et al., 2015
Chitosan/alginate (Chi/alg) NPs	Chitosan (Mv^f^ of 1,10,000–1,50,000) Sodium alginate (very low viscosity)	1:10^g^ 300 nm, −30 mV (water) 900 nm, −25 mV (cell culture medium) 10:1^g^ 500 nm, + 30 mV (water) 1,100 nm, + 10 mV (cell culture medium)	*In vivo* exposure (14 days)	Wistar albino rats	Oral	9 mg/kg b.w. (in 0.5 ml/100 g b.w.)	No mortality No behavioral changes No changes in body weight or relative liver weight No changes in MDA levels GSH levels decreased for the 10:1 (chit:alg) ratio No hematological parameters altered	Aluani et al., 2017
Chitosan/alginate (Chi/alg) NPs	Chitosan (low molecular weight; 200 cp viscosity) Sodium Alginate (low viscosity −0.02 Pa.s)	1:9^g^ 254 nm, −35 mV	*In vivo* exposure (14 days)	Wistar albino rats	Oral	24.5 mg (in 2 mL)	No mortality No adverse reaction in the condition of the eye, nose and motor activity No histopathological alteration in animal's organs Normal feed intake and weight gain	Radwan et al., 2017b
pH sensitive chitosan/poly-γ-glutamic acid (Chi/PGA) NPs	Chitosan (80 kDa, 85% DD) γ -PGA (60 kDa)	218 nm +25.3 mV	*In vivo* exposure (14 days)	ICR mice	Oral	100 mg/kg b.w.	No clinical signs or weight loss No change in hematological or biochemical parameters No pathological changes in liver, kidney and intestinal segments *The dose (100 mg/kg) was 18 times higher than the dose they used in the pharmacokinetic study of insulin-loaded nanoparticles (5.5 mg/kg)*	Sonaje et al., 2009
α-tocopherol succinate-grafted carboxymethyl chitosan polymeric micelles	low molecular weight chitosan: 22 kDa	114–187 nm −20 to −22 mV	*In vivo* exposure (14 days)	Sprague Dawley rats	Oral	500 mg/kg b.w.	No mortality Normal weight gain Normal red blood cells morphology No pathological changes in the liver, kidney, and intestine	Jena and Sangamwar, 2016
Alginate coated CS core-shell NPs	Sodium alginate (ALG) of low viscosity, ~50 kDa Low molecular weight CS (25 kDa, DDA 82%)	216 nm −36 mV (with naringenin encapsulated)	*In vivo* exposure (19 days)	Wistar rats	Oral	50 mg/kg b.w. (blank NPs)	No significant differences in hair texture or color, water and food intake No hepatic toxicity No abnormalities found in the hepatic or intestinal tissues No hematological parameters change (glucose and lipids)	Maity et al., 2017
Oleoyl-carboxymethyl-chitosan (OCMCS) nanoparticles	170 kDa chitosan, 92.56% DD modified with chloroactic acid and oleoyl chloride	171 nm + 19 mV	*In vivo* exposure (7 days)	Carp	Oral (catheter)	2 mg/mL (500 μL)	No lethality or histopathological signs of inflammation (liver, spleen, kidneys)	Liu et al., 2013
Amphotericin loaded PEG-PLGA NPs	PLGA lactic to glycolic acid 50:50 with 40–75 KDa and PEG with 10 KDa	170 nm	*In vivo* exposure (7 days)	Wistar rats	Intraperitoneal and oral	Equivalent to 10 mg/kg b.w.of amphotericin	No lethality, no body weight loss, no hematological parameters alterations, no histopathological changes in liver, and kidneys	Moraes Moreira Carraro et al., 2017
Amphotericin loaded PLGA NPs	PLGA lactic to glycolic acid 50:50 with 40–75 KDa	190 nm						
Chitosan/alginate (Chi/alg) NPs	Chitosan (Mv^h^ of 1,10,000–1,50,000) Sodium alginate (very low viscosity)	1:10^i^ 300 nm, −30 mV (water) 900 nm, −25 mV (cell culture medium) 10:1^i^ 500 nm, + 30 mV (water) 1,100 nm, + 10 mV (cell culture medium)	*In vivo* exposure (14 days)	Wistar albino rats	Oral	9 mg/kg b.w. (in 0.5 ml/100 g b.w.)	No mortality No behavioral changes No changes in body weight or relative liver weight No changes in MDA levels GSH levels decreased for the 10:1 (chit:alg) ratio No hematological parameters altered	Aluani et al., 2017
Chitosan/alginate (Chi/alg) NPs	Chitosan (low molecular weight; 200 cp viscosity) Sodium Alginate (low viscosity −0.02 Pa.s)	1:9^i^254 nm, −35 mV	*In vivo* exposure (14 days)	Wistar albino rats	Oral	24.5 mg (in 2 mL)	No mortality No adverse reaction in the condition of the eye, nose, and motor activity No histopathological alteration in animal's organs Normal feed intake and weight gain	Radwan et al., 2017b
pH sensitive chitosan/poly-γ-glutamic acid (Chi/PGA) NPs	Chitosan (80 kDa, 85% DD) γ -PGA (60 kDa)	218 nm +25.3 mV	*In vivo* exposure (14 days)	ICR mice	Oral	100 mg/kg b.w.	No clinical signs or weight loss No change in hematological or biochemical parameters No pathological changes in liver, kidney, and intestinal segments *The dose (100 mg/kg) was 18 times higher than the dose they used in the pharmacokinetic study of insulin-loaded nanoparticles (5.5 mg/kg)*	Sonaje et al., 2009
Dissulfiram loaded PLGA nanoparticles, coated with PEG and functionalized with folate	PLGA (RG 504 H, acid terminated, lactide:glycolide 50:50, Mw: 38,000) and PEG-bis-amine (Mn: 10,000)	204 nm −5.24 mV	*In vivo* exposure (7 days)	BALB/C mice	Intravenous	Equivalent to 120, 60, 30, and 15 mg/kg of dissulfiram 120 mg/kg b.w. blank nanoparticles	No lethality, no hematological parameters changes *(2,000 mg/kg of loaded NPs ~100 mg/kg equivalent of disulfiram)*	Fasehee et al., 2016
Polyphenolic bio-enhancers with oleanolic acid in chitosan coated PLGA NPs (CH-OA-B-PLGA NPs)	chitosan (molecular weight 150 kDa, deacetylation degree 85%), Poly (lactide-coglycolide) (PLGA) 50:50, mw 40–75 kDa	342 nm + 34 mV	*In vivo* exposure (15 days)	Sprague Dawley rats	Oral	100 mg/kg b.w. of OA	No mortality No histopathological changes No abnormal behavior *(100 mg/kg is the double of the OA effective dose)*	Sharma et al., 2017
Polyphenolic bio-enhancers with oleanolic acid in PLGA NPs (OA-B-PLGA NPs)	chitosan (molecular weight 150 kDa, deacetylation degree 85%), Poly (lactide-coglycolide) (PLGA) 50:50, mw 40–75 kDa	221 nm −19 mV	*In vivo* exposure (15 days)	Sprague Dawley rats	Oral	100 mg/kg b.w. of OA	No mortality No histopathological changes No abnormal behavior *(100 mg/kg is the double of the OA effective dose)*	Sharma et al., 2017
Amphotericin loaded PEG-PLGA nanoparticles	Copolymer produced with 6,000 Da PLGA (lactic to glycolic acid molar ratio of 1:1) and 15% PEG	25 nm	*In vivo* exposure (7 days)	Albino Sprague-Dawley rats	Intravenous	Equivalent to 1 mg/kg of amphotericin and blank NPs	No nephrotoxicity (evaluated by renal injury biomarkers BUN and PCr) No histopathological damage of the kidney Although described no results presented for blank nanoparticles group	Radwan et al., 2017a
Paclitaxel loaded monomethoxypoly (ethylene glycol)-b-poly(lactic acid) (mPEG-PLA) polymeric micelles	mPEG-PLA copolymer (40/60) with a number average molecular weight of 4488.4	(40/60): 37 nm After incubation with BSA: 40 nm (50/50): 44 nm After ncubation with BSA: 71 nm	*In vivo* exposure (4 weeks, 1 injection per week)	Beagle dogs	Injection in the foreleg (intravenous)	Equivalent to 0.5 mg/mL of paclitaxel	mPEG-PLA (40/60): no sign of pathological changes except the lung congestion. mPEG-PLA (50/50): liver index was higher and the thymus index was lower;pylorus and small intestine congestion were also observed The toxicity of paclitaxel loaded mPEG-PLA (40/60) polymeric micelles was significantly lower than those of mPEG-PLA (50/50)	Li et al., 2014
Angiopoietin-2 (Ang2) small interfering (si)RNA plasmid chitosan magnetic nanoparticles (CMNPs)	Chitosan polysaccharides (Mw^j^ 13,80,000, 90% DD)	na^e^	*In vivo* exposure (14 days)	Sprague-Dawley rats	Intravenous	35, 70, and 353 mg/kg b.w.	Higher doses: chronic pulmonary congestion in Sprague-Dawley rats, as well as simultaneous pulmonary inflammation and partial fibrosis All doses: total number of white blood was significantly higher *Based on the conversion method of equivalent dose co-efficient, the non-toxic dose in humans should be <222 mg/kg per day for 14 day, overall a total of 3,117 mg/kg, which is significantly higher compared with the quantity required clinically*	Shan et al., 2017

a *DD, deacetylation degree*.

b *M/G, β-D-mannuronic acid/α-L-guluronic acid*.

c *Ratio alg:chi:ins*.

d *Mw, molecular weight number*.

e *na, not available*.

f *Mv, viscosity molecular weight*.

g *Ratio chi:alg*.

As already stated, chitosan NMs are the most studied polymeric NMs regarding toxicity. Several studies were found in the literature evaluating the toxicity of blend chitosan NPs upon repeated oral administrations. Despite the great heterogeneity among the used NPs (chitosan/alginate NPs, chitosan/glutamic acid NPs, oleoyl-carboxy methyl chitosan NPs, chitosan coated PLGA NPs and α-tocopherol succinate-g-carboxymethyl chitosan NPs), the animal models (Wistar and Sprague Dawley rats, ICR mice and Carps) and the dosing schedules (7–19 days), all revealed no *in vivo* toxicity (Sonaje et al., 2009; Liu et al., 2013; Jena and Sangamwar, 2016; Aluani et al., 2017; Maity et al., 2017; Radwan et al., 2017b; Sharma et al., 2017). Moreover, the conclusion of no toxicity was based on different evaluated parameters for each study, except for the histopathological analysis, which was performed in all studies (generally liver and intestine histopathology with no signs of tissue damage). Among these studies, only Sonaje et al. (2009), Maity et al. (2017), and Radwan et al. (2017b) have evaluated biochemical parameters in blood, and in common have tested serum alanine transaminase (ALT), alkaline phosphatase (ALP) and aspartate transaminase (AST) activities, and their results were in agreement (no changes in comparison to the control group). Moreover, chitosan based NPs lack of oral toxicity was also reported for single dose administrations (Mukhopadhyay et al., 2015; Leng et al., 2018). Therefore, considering these reports, we may hypothesize that chitosan NPs (as well as bulk chitosan Chang et al., 2014) do not present oral toxicity. On the other hand, although only 2 reports were found testing chitosan NPs toxicity through the injectable route (Yuan et al., 2015; Shan et al., 2017), a dose dependent toxicity was found, even though chitosan and chitosan NPs appear to be hemocompatible in some hemolysis assays (Fernandes et al., 2010; Lü et al., 2011; Wang et al., 2014; Kumar et al., 2017; Leng et al., 2018).

On its turn, PLGA NPs also exhibited no toxicity on repeated oral administration studies (Moraes Moreira Carraro et al., 2017; Sharma et al., 2017), as well as on the majority of intravenous (i.v.) administration studies (VasanthaKumar et al., 2014; Fasehee et al., 2016; Radwan et al., 2017a). Only one article described some toxicity when using danorubicin loaded PEG-PLL-PLGA NPs (Guo et al., 2015). Unfortunately, the formulations in those reports were loaded with the active drug and no information was given on blank NPs. Therefore, not only the effects might be associated with the drugs (rather than the NPs polymers or characteristics), but also no comparison on the dose of the NPs administered can be made between articles, as they only refer to the equivalent amount of drug administered. Similarly (Li et al., 2014), tested two mPEG-PLA NPs (with different copolymerization degrees) loaded with paclitaxel in beagle dogs by i.v. administration in the foreleg. Despite the results had revealed differences between the NPs, being the ones with the 50/50 ratio mPEG:PLA more toxic than the ones with the 40/60, no experiments were made with unloaded NPs, restricting the extrapolation of data.

### Oxidative Stress

Reactive oxygen species (ROS) are produced during cellular metabolism in the forms of hydrogen peroxide (H_2_O_2_), superoxide anion (O2^−•^) and hydroxyl (^•^OH) radicals (Ngo and Kim, 2014; Lorscheidt and Lamprecht, 2016). Besides its role in cell signaling and regulation, excessive oxidative stress can induce oxidative damage to cells through lipid peroxidation, DNA disruption, interference with signaling functions, gene transcription modulation and inadvertent enzyme activation, causing several health disorders, such as hypertensive, cardiovascular, inflammatory, aging, diabetes mellitus, and neurodegenerative and cancer diseases (Sharifi et al., 2012; Ngo and Kim, 2014; Lorscheidt and Lamprecht, 2016).

The most used probe to access ROS is the H_2_O_2_ specific 2′,7′-dichlorodihydrofluorescein diacetate (H_2_DCFDA or DCFH-DA), which diffuses freely through the cell membrane and is hydrolyzed inside the cells into H_2_DCF carboxylate anion form, which is in its turn non-permeable (Kalyanaraman et al., 2012; Oparka et al., 2016). Then, H_2_DCF is oxidized and results in the formation of the fluorescent product (DCF), which is excited at 495 nm and emits at 520 nm (Kalyanaraman et al., 2012; Oparka et al., 2016). Using this probe, the intracellular signal can be monitored by several techniques, such as confocal microscopy and flow cytometry (Kalyanaraman et al., 2012). During the H_2_DCF oxidation, there is a formation of a superoxide radical that can stimulate the auto-amplification of the DCF signal (Oparka et al., 2016). On the other hand, DCF is cell permeable, which means it leaks out of cells over time and can induce measurement errors depending on the analysis time (Lorscheidt and Lamprecht, 2016). A variant of the DCFH-DA probe is the 5-(and 6)-chloromethyl-derivative, that leads to the formation of fluorescent CM-DCF, which displays a lower passive leakage from the cell (Oparka et al., 2016). Alternatively, the fluorescence read-out can also be performed using a fluorescence microplate reader and in this situations errors can result from nanoparticle quenching effect over the DCF fluorescence (Aranda et al., 2013).

Free radical production is the highest in macrophages (Singh and Ramarao, 2013) which is in line with the protocol suggested in ISO/TS 19006:2016-Nanotechnologies-5-(and 6)-Chloromethyl-2′,7′-Dichloro-dihydrofluorescein diacetate (CM-H_2_DCF-DA) assay for evaluating nanoparticle-induced intracellular reactive oxygen species (ROS) production in RAW 264.7 macrophage cell line. Nonetheless, according to this ISO, other cell lines similar to RAW 264.7 (BEAS-2B, RLE-6TN, HEPA-1, HMEC and A10) can be used with due validations. In this technical specification, the protocol was validated for conducting the assay in 24 well-plates, for 6 and 24 h incubation with the NPs and controls, and 30 min incubation with the probe before flow cytometry analysis. To note, the recommendation is the use of Sin-1 as positive control (maximum ROS production due to cell death) and polystyrene NPs as negative control.

As it is possible to observe from Table 4, most studies reported in the literature do not use RAW 264.7 cells, neither do they employ 6 and 24 h incubation.

**Table 4:** Review of original articles assessing oxidative stress induction by polymeric nanoparticles.

**Nanomaterial**	**Polymer characterization**	**Nanomaterial characterization**	**Testing method**	**Cellular model**	**Dose/concentration range**	**Results**	**Observations**	**References**
Chitosan NPs	Low molecular weight chitosan (50–190 kDa, 75–85% DD^a^)	92 nm +32 mV	2′,7′-dichlorodihydro- fluorescein diacetate (H_2_DCF-DA) probe (72 h incubation)	HeLa, MDA-MB-231 and THP-1 cells	1%	Significant reduction in the generation of reactive oxygen species when compared to control	Similar results for plasmid loaded chitosan NPs	Bor et al., 2016
Chitosan NPs	80% DD 400 kDa	100 nm + 19 mV	Dichlorofluorescin diacetate (DCFH-DA) probe (6/12/24 h incubation)	Hela and SMMC-7721 cells	10; 100 μg/mL	Chitosan NPs increase ROS production in a concentration-dependent manner	–	Wang et al., 2018
Chitosan NPs	Low molecular weight chitosan (85% DD)	≤ 100 nm + 40 mV	Dichlorofluorescin diacetate (DCFH-DA) probe (unknown h incubation)	BCL2(AAA) Jurkat cells	10–50 μg/mL	All concentrations induced ROS production (concentration dependent manner)	Bulk chitosan was tested at the same concentrations. ROS production was concentration dependent but lower than with chitosan NPs	Sarangapani et al., 2018
Chitosan NPs	na	164 nm; + 63 mV 385 nm; + 62 mV 459 nm; +72 mV 475 nm; +71 mV 685 nm; +74 mV	Dihydroethidium (DHE) probe (72 h incubation)	Mouse bone marrow-derived hematopoietic stem cells	250–1,000 μg/mL	ROS production was not significantly altered following exposure to chitosan NPs	–	Omar Zaki et al., 2015
Chitosan NPs	75–85% 50–190 kDa	173 nm + 23 mV	Dichlorofluorescin diacetate (DCFH-DA) probe (24 h incubation)	HEK-293 cells	100 μg/mL	Chitosan NPs had no effect on ROS production	Bulk chitosan was also tested and had no effect in ROS production	Arora et al., 2016
PLA NPs	Poly(D,L-lactide) (PDLLA) 1,01,782 g/mol and 0.68 dL/g	188 nm −24 mV (water) 78 nm −0.4 mV (DMEM^b^)	2′,7′-Dichlorofluorescin diacetate (DCFH-DA) probe (24 h incubation)	RAW 264.7 cells	4.3, 17, 34, 340 μg/mL	PLA NPs with 78 nm in DMEM caused a significant increase in ROS production for the highest concentration tested (340 μg/mL)	The increase in ROS production was related to cytotoxicity. The sample and concentration that induced ROS production decreased cell viability to values close to 70%. All the other concentrations were close to 100%	Da Silva et al., 2019
PLA NPs	Poly(D,L-lactide) (PDLLA) 1,01,782 g/mol and 0.68 dL/g	109 nm −7 mV (water) 154 nm −0.7 mV (DMEM)	2′,7′-Dichlorofluorescin diacetate (DCFH-DA) probe (24 h incubation)	RAW 264.7 cells	8.6, 34, 69, 690 μg/mL	No ROS production observed	–	Da Silva et al., 2019
PLA NPs	na	176 nm −58 mV In cell culture: 212 nm −24 mV	2′,7′-Dichlorofluorescin diacetate (DCFH-DA) probe (72 h incubation)	Schneider's *Drosophila melanogaster* line 2 (S2) cells	0.5–500 μg/mL	ROS production was only observed at the highest tested concentration (500 μg/mL) indicating a concentration dependent effect	–	Legaz et al., 2016
PLGA NPs	Resomer^®^ RG503H, acid terminated, 50:50, Mw 24,000–38,000	80 nm −25 mV	2′,7′-Dichlorofluorescin diacetate (DCFH-DA) probe (3 h incubation)	16HBE14o-, L5178Y, and TK6 cells	40 μg/mL	No increase in ROS production in 16HBE14o-, L5178Y, and TK6 cells, in comparison to the control	The L5178Y mouse lymphoma and TK6 human B-lymphoblastoid cells, are routinely used in *in vitro* regulatory genotoxic assays. The human bronchial epithelial cells 16HBE14o-, a cell line is suitable for toxicity studies of inhaled NPs as it is highly similar to the primary bronchial epithelium	Platel et al., 2016
hexadecyltrimethylammonium bromide (CTAB) stabilized PLGA NPs	Resomer^®^ RG503H, acid terminated, 50:50, Mw 24,000–38,000 and PEG 2,000	82 nm +15 mV	2′,7′-Dichlorofluorescin diacetate (DCFH-DA) probe (3 h incubation)	16HBE14o-, L5178Y, and TK6 cells	40 μg/mL	Significant increase in ROS production in 16HBE14o-, L5178Y, and TK6 cells, in comparison to the control	The L5178Y mouse lymphoma and TK6 human B-lymphoblastoid cells, are routinely used in *in vitro* regulatory genotoxic assays. The human bronchial epithelial cells 16HBE14o-, a cell line is suitable for toxicity studies of inhaled NPs as it is highly similar to the primary bronchial epithelium	Platel et al., 2016
Polyphenolic bio-enhancers with oleanolic acid in chitosan coated PLGA NPs (CH-OA-B-PLGA NPs)	Chitosan (molecular weight 150 kDa, deacetylation degree 85%), Poly (lactide-coglycolide) (PLGA) 50:50, mw 40–75 kDa	342 nm + 34 mV	2′,7′-Dichlorofluorescin diacetate (DCFH-DA) probe (24 h incubation)	MDAMB-231 cells	na	Increased proxidant effect of CH-OA-B-PLGA was two times higher than plain OA	100 mg/kg is the double of the OA effective dose	Sharma et al., 2017
Poly-lactic-co-glycolic acid–polyethylene oxide (PLGA–PEO) NPs	(Purchased from Advancell)	140 nm −43 mV (in cell culture medium)	Hydroethidine probe (24–48 h incubation)	16HBE14o- and A549 cells	37.5 and 75 μg/cm^2^	Weak production of intracellular ROS at the highest concentrations used, only in the A549 cell line	–	Guadagnini et al., 2013b
PLGA NPs	75:25 Resomer^®^ RG756	170 nm −45 mV (200 nm in cell culture medium)	2′,7′-Dichlorofluorescin diacetate (DCFH-DA) probe (5 min−48 h incubation)	THP-1 cell culture	0.1 or 1 mg/mL	No Induction of ROS production at 0.1 mg/mL At 1 mg/mL, a transient increase in ROS production was verified at 5 min	THP-1 monocytes differentiation into macrophages was performed using 12-o-tetradecanoylphorbol-13-acetate (PMA)	Grabowski et al., 2015
PVA stabilized PLGA NPs	75:25 Resomer^®^ RG756 and PVA (87–89% hydrolyzed, 30–70 kDa)	Ratio PVA:PLGA 11.5:100 230 nm −1 mV (210 nm in cell culture medium)	2′,7′-Dichlorofluorescin diacetate (DCFH-DA) probe (5 min−48 h incubation)	THP-1 cell culture	0.1 or 1 mg/mL	No Induction of ROS production at 0.1 mg/mL At 1 mg/mL, a transient increase in ROS production was verified at 5 min	THP-1 monocytes differentiation into macrophages was performed using 12-o-tetradecanoylphorbol-13-acetate (PMA)	Grabowski et al., 2015
Chitosan stabilized PLGA NPs	75:25 Resomer^®^ RG756 and Protasan^®^ UP CL113, 75–90% deacetylation, 50–150 kDa	Ratio chi:PVA:PLGA 15.3:30.4:100 230 nm + 40 mV (270 nm in cell culture medium)	2′,7′-Dichlorofluorescin diacetate (DCFH-DA) probe (5 min−48 h incubation)	THP-1 cell culture	0.1 or 1 mg/mL	No Induction of ROS production at 0.1 mg/mL At 1 mg/mL, a transient increase in ROS production was verified at 5 min	THP-1 monocytes differentiation into macrophages was performed using 12-o-tetradecanoylphorbol-13-acetate (PMA)	Grabowski et al., 2015
Pluronic stabilized PLGA NPs	75:25 Resomer^®^ RG756 and Pluronic F68	Ratio F68:PLGA 15.5:100 230 nm −30 mV (315 nm in cell culture medium)	2′,7′-Dichlorofluorescin diacetate (DCFH-DA) probe (5 min−48 h incubation)	THP-1 cell culture	0.1 or 1 mg/mL	No Induction of ROS production at 0.1 and 1 mg/mL	THP-1 monocytes differentiation into macrophages was performed using 12-o-tetradecanoylphorbol-13-acetate (PMA)	Grabowski et al., 2015
PLGA NPs	50:50^c^ (intrinsic viscosity 0.60 g/dl) 65:35^c^ (intrinsic viscosity 0.64 g/dl) 75:25^c^ (intrinsic viscosity 0.72 g/dl) 85:15^c^ (intrinsic viscosity 0.62 g/dl)	210 nm −14 mV 211 nm −8.70 mV 218 nm −12.7 mV 243 nm −12.7 mV	2′,7′-Dichlorofluorescin diacetate (DCFH-DA) probe (24 h incubation)	RAW 264.7 cells	10, 30, 100, and 300 μg/mL	No effect on ROS production up to 100 μg/ml concentration; 300 μg/ml showed 1.5- to 2-fold stimulation of ROS production A further increase in NPs concentration to 1,000 μg/ ml interfered with ROS assay due to fluorescence quenching	No significant differences were found in these assays between these NPs	Singh and Ramarao, 2013
PLA NPs	DL-PLA (MW 10,000)	256 nm −17.1 mV	2′,7′-Dichlorofluorescin diacetate (DCFH-DA) probe (24 h incubation)	RAW 264.7 cells	10, 30, 100, and 300 μg/mL	No effect on ROS production up to 100 μg/ml concentration; 300 μg/ml showed 1.5- to 2-fold stimulation of ROS production A further increase in NPs concentration to 1,000 μg/ ml interfered with ROS assay due to fluorescence quenching	–	Singh and Ramarao, 2013
PCL NPs	PCL (intrinsic viscosity 1.07 g/dl)	268 nm −9.10 mV	2′,7′-Dichlorofluorescin diacetate (DCFH-DA) probe (24 h incubation)	RAW 264.7 cells	10, 30, 100, and 300 μg/mL	No effect on ROS production up to 100 μg/ml concentration; 300 μg/ml showed 1.5- to 2-fold stimulation of ROS production A further increase in NPs concentration to 1,000 μg/ ml interfered with ROS assay due to fluorescence quenching	–	Singh and Ramarao, 2013
Poly(lactide-co-caprolactone) (PLCL) NPs	PLCL 25:75 (intrinsic viscosity 0.71 g/dl) PLCL 80:20 (intrinsic viscosity 0.77 g/dl	261 nm −15.3 mV 261 nm −15.4 mV	2′,7′-Dichlorofluorescin diacetate (DCFH-DA) probe (24 h incubation)	RAW 264.7 cells	10, 30, 100, and 300 μg/mL	No effect on ROS production up to 100 μg/ml concentration; 300 μg/ml showed 1.5- to 2-fold stimulation of ROS production A further increase in NPs concentration to 1,000 μg/ ml interfered with ROS assay due to fluorescence quenching	–	Singh and Ramarao, 2013

a *DD, deacetylation degree*.

b *DMEM, Dulbecco's Modified Eagle Medium*.

c *PLGA lactic to glycolic acid*.

In detail, Grabowski et al. found a transient production of ROS with chitosan stabilized PLGA NPs in THP-1 cells (Grabowski et al., 2015), Sharma et al. verified an increased oxidative effect of oleanolic acid when delivered by chitosan coated PLGA NPs in MDAMB-231 cells (Sharma et al., 2017), Sarangapani et al. found an increase in ROS production in BCL2(AAA) Jurkat cells with chitosan NPs (Sarangapani et al., 2018) and Gao et al. found an increase in ROS production in zebrafish embryos incubated with chitosan NPs (Hu et al., 2011). In contrast, Bor et al. found a reduction in ROS production with plasmid loaded chitosan NPs and chitosan NPs in Hela, THP-1 and MDAMB-231 cells (Bor et al., 2016). These inconsistent results, obtained with different chitosan based nanomaterials, different cellular models and concentrations do not allow for a straightforward interpretation of the oxidative effect of nanoscale chitosan. Among these articles, only Sarangapani et al. compared the activity of chitosan NPs with bulk chitosan (at the same concentrations) and verified a similar but lower concentration dependent effect for the polymer (Sarangapani et al., 2018). Also, it is important to note, that the tested concentrations (10–50 μg/mL), caused increasing cell death as verified by the MTT assay, and therefore, the oxidative stress was the mechanism identified as responsible for cellular toxicity. In contrast, Bor et al. verified that chitosan NPs reduced ROS production in several cell lines (also tumor derived cells), but they used a concentration that did not cause cell death (Bor et al., 2016). Therefore, although at first sight the results are conflicting, they cannot be directly compared, but we can hypothesize that chitosan NPs might influence ROS production in a concentration dependent manner. One of the widely reported characteristics of bulk chitosan is its anti-oxidant activity, attributed to its scavenging activity against several radicals, such as hydroxyl (^•^OH), superoxide anion (${\text{O}}_{2}^{•-}$), 1,1-diphenyl-2-picryl-hydrazy (DPPH) and alkyl (Ngo and Kim, 2014). This scavenging activity, has been widely demonstrated by cell-free *in vitro* assays (Je et al., 2004; Yen et al., 2008; Ngo and Kim, 2014). In fact, in the article discussed before (Sarangapani et al., 2018), although reporting that chitosan and chitosan NPs increased ROS production in BCL2(AAA) Jurkat cells, they also verified that the same concentrations increased free radical scavenging activity using chemical assays. Therefore, some compounds may demonstrate chemically some antioxidant activity, which is not verified at cellular and physiological level (Lü et al., 2010).

Regarding bare PLGA NPs its effect on ROS production was documented by 3 authors Platel, Singh, and Granbowski (Singh and Ramarao, 2013; Grabowski et al., 2015; Platel et al., 2016) all using different cellular models. Nevertheless, Platel tested only one low concentration of PLGA NPs (40 μg/mL) and found no effect on ROS production (Platel et al., 2016), while the other 2 authors found an increase in ROS production that was dose dependent (Singh and Ramarao, 2013; Grabowski et al., 2015). Curiously, both tested 1 mg/mL, but Singh et al. reported that this concentration quenched the fluorescence of the probe, therefore interfering with the results (Singh and Ramarao, 2013). On its turn, Grabowski et al. found that at the concentration of 1 mg/mL only a transient production of ROS was verified at 5 min after the incubation with PLGA NPs, and at longer incubation times, no significant ROS increase was verified (Grabowski et al., 2015). Although the authors do not explore this achievement, we could hypothesize that a similar interference as reported by Singh and Ramarao might be occurring.

Overall, not only PLGA NPs, but in general the polyester NPs appear to induce ROS production in a concentration dependent manner. Other studies confirm this effect for concentrations above 300 μg/mL (Singh and Ramarao, 2013; Legaz et al., 2016; Da Silva et al., 2019). Nevertheless, this conclusion has reservations since for instance, Da Silva et al. tested two different PLA NPs, and only one of these induced ROS production.

### Inflammation

Presently, inflammation is acknowledged as a mechanism of immune defense and repair, in addition to its widely accepted role in passive cell injury and cell death (Wallach et al., 2013; Khanna et al., 2015). Interestingly, several molecules are associated with inflammation and cell death. For instance TNF-α, IL-1β, IL-6, IFN-γ, IL-17, IL-8, IL-2, GM-CSF, TGF-β, and IL-12 are examples of pro-inflammatory mediators frequently evaluated in the context of cellular toxicity induced by nanomaterials (Khanna et al., 2015; Lorscheidt and Lamprecht, 2016).

Regarding the methodologies, the enzyme-linked immunosorbent assay (ELISA) is widely applied as a simple mean to perform a qualitative and quantitative analysis of cytokines, chemokines, growth factors and immunoglobulins, with a spectrophotometric readout (Lorscheidt and Lamprecht, 2016). In this assay, the pro- and anti-inflammatory mediators are released into cell supernatant, which is collected and then analyzed. Therefore, the release of cytokines or other molecules by cells during the incubation with nanoparticles can be underestimated due to the nanoparticles ability to adsorb biomolecules at its surface (Lorscheidt and Lamprecht, 2016). Kroll et al. (2012) tested the potential interference of 4 types of engineered nanoparticles on IL-8 secretion, and verified that a specific pre-dispersion of TiO_2_ nanoparticles was able to reduce the measurable levels of the cytokine, under the assay conditions. Similarly, Guadagnini et al. (2013a), tested 4 types of nanoparticles in acellular conditions and verified that TiO_2_, SiO_2_, and Fe_3_O_4_ NPs decreased the cytokines levels due to surface adsorption. In the same experiment, PLGA-PEO NPs induced an apparent increase in GM-CSF levels, which the authors believe may be due to the stabilization of the peptides, their protection from proteolysis or by avoiding the interaction of this cytokine with the plastic of the culture plates (Guadagnini et al., 2013a). Although most of the reported interferences are for inorganic nanoparticles, these are good examples that can be overlooked when performing ELISA in cell supernatants previously incubated with polymeric nanoparticles. When studying pro- and anti-inflammatory molecules release due to NPs stimulation, it can be useful to previously study the adsorption or interaction of the NPs with the molecules (i.e., cytokine standards) in acellular conditions.

Alternatively, instead of measuring cell secreted pro- and anti-inflammatory molecules by ELISA, the mRNA levels inside the cell can be measured with RT-qPCR (Real-Time quantitative Polymerase Chain Reaction) or the intracellular levels of the cytokines can be measured by flow cytometry analysis using specific antibodies fluorescently labeled (Lorscheidt and Lamprecht, 2016). In the first alternative, however, an increase of mRNA expression does not necessarily lead to an increase of protein secretion (Guadagnini et al., 2013a).

Lastly, besides the masking/enhancing effect of NPs, the presence of contaminants, such as endotoxins can induce itself increased levels of pro-inflammatory molecules in cells (Oostingh et al., 2011). Endotoxins, commonly referred to as lipopolysaccharide (LPS), are present in the outer cell membrane of Gram negative bacteria and are released during multiple processes, such as cell death, growth and division (Magalhaes et al., 2007; Lieder et al., 2013). Therefore, due to the bacteria ability to growth and adapt in several environments, LPS is easily found in numerous media, including poor nutrient media (water, saline and buffers) and its removal is a struggle since it is highly resistant to extreme temperatures pHs (Magalhaes et al., 2007). LPS is comprised by a O-antigen region, a hydrophilic core oligosaccharide and a hydrophobic Lipid A (LipA) (Davydova et al., 2000; Magalhaes et al., 2007; Steimle et al., 2016). The lipid A structure, highly conserved, differs among bacterial species, and determines the molecule immunogenicity (Steimle et al., 2016). On the whole, LPS is a pathogen associated molecular pattern (PAMP), which is recognized and activates the mammalian innate immune system, leading for instance to cellular release of pro-inflammatory cytokines and free radicals, particularly by monocytes and macrophages (Yermak et al., 2006; Lieder et al., 2013; Steimle et al., 2016). Consequently, *in vitro* testing of LPS contaminated polymeric NMs might generate misleading results and false assumptions of bioactivity or toxicity, ultimately affecting the evaluation of possible human health effects (Lieder et al., 2013).

Table 5 summarizes the results found in the literature for polymeric NPs stimulation of cytokines.

**Table 5:** Review of original articles assessing inflammatory cytokines induced by polymeric nanoparticles in different cells.

**Nanomaterial**	**Polymer characterization**	**Nanomaterial characterization**	**Testing method**	**Cellular model**	**Dose/concentration range**	**Results**	**Endotoxin contamination**	**References**
Chitosan NPs	95 ± 20 kDa	290 nm +37 ± 1.4	*In vitro* cytokine production (24 h incubation) (IL-1β, IL-6, TNF-α, MCP-1α, and MIP-1)	RAW 264.7 and BMDCs	–	RAW 264.7: production of MIP1 and TNF-α, IL6, and MCP1 but not of IL-1β BMDCs: production of MIP1, TNF-α, IL-1β, IL6, and MCP1^a^	–	Koppolu and Zaharoff, 2013
Chitosan NPs	50–190 KDa	70 nm + 15 mV	*In vitro* cytokine production (30 min incubation + 24 h) (IL-1β, IL-6, IL-12p70, and TNF-α)	BMDCs	–	No cytokine production	–	Han et al., 2016
Poly-lactic-co-glycolic acid–polyethylene oxide (PLGA–PEO) NPs	(Purchased from advancell)	140 nm −43 mV (in cell culture medium)	*In vitro* cytokine production (24–48 h incubation) (GM-CSF, IL-6, IL-8, IL-1β)	16HBE14o- and A549 cells	75 μg/cm^2^	No significant increase of any cytokine mRNA after 24 or 48 h Interestingly, there was a decreased level of all cytokine mRNA in A549 cells after PLGA-PEO NP exposure	mRNA cytokine analysis was performed through RT-qPCR	Guadagnini et al., 2013b
PLGA NPs	75:25 Resomer^®^ RG756	170 nm −45 mV (200 nm in cell culture medium)	*In vitro* cytokine production (24 h incubation) (IL-8, IL-6, TNF-α, and MCP-1)	A549 and THP-1-D cell co-culture	0.1 or 1 mg/mL	0.1 mg/mL did not induce cytokine secretion 1 mg/mL induced IL-6, TNF-α and MCP-1^16^	Endotoxin (LPS) determination was performed in the supernatant (12,000 g, 30′) of all formulations diluted in cell culture medium for the used *in vitro* concentrations with LAL chromogenic endotoxin quantitation kit. Results showed endotoxin values between 0.1 and 0.3 EU/mL.	Grabowski et al., 2016
PVA stabilized PLGA NPs	75:25 Resomer^®^ RG756 and PVA (87–89% hydrolyzed, 30–70 kDa)	230 nm −1 mV (210 nm in cell culture medium)	*In vitro* cytokine production (24 h incubation) (IL-8, IL-6, TNF-α, and MCP-1)	A549 and THP-1-D cell co-culture	0.1 or 1 mg/mL	0.1 mg/mL induced IL-8 and MCP-1 1 mg/mL induced IL-6^b^	Endotoxin (LPS) determination was performed in the supernatant (12,000 g, 30′) of all formulations diluted in cell culture medium for the used *in vitro* concentrations with LAL chromogenic endotoxin quantitation kit. Results showed endotoxin values between 0.1 and 0.3 EU/mL.	Grabowski et al., 2016
Chitosan stabilized PLGA NPs	75:25 Resomer^®^ RG756 and Protasan^®^ UP CL113, 75–90% deacetylation, 50–150 kDa	230 nm +40 mV (270 nm in cell culture medium)	*In vitro* cytokine production (24 h incubation) (IL-8, IL-6, TNF-α and MCP-1)	A549 and THP-1-D cell co-culture	0.1 or 1 mg/mL	0.1 mg/mL induced IL-8 and MCP-1 1 mg/mL induced IL-6 and MCP-1^16^	Endotoxin (LPS) determination was performed in the supernatant (12,000 g, 30′) of all formulations diluted in cell culture medium for the used *in vitro* concentrations with LAL chromogenic endotoxin quantitation kit. Results showed endotoxin values between 0.1 and 0.3 EU/mL.	Grabowski et al., 2016
Pluronic F68 stabilized PLGA NPs	75:25 Resomer^®^ RG756 and Pluronic PF68 (BASF)	230 nm −30 mV (315 nm in cell culture medium)	*In vitro* cytokine production (24 h incubation) (IL-8, IL-6, TNF-α, and MCP-1)	A549 and THP-1-D cell co-culture	0.1 or 1 mg/mL	0.1 mg/mL induced MCP-1 1 mg/mL induced il-8. Il-6 and MCP-1^16^	Endotoxin (LPS) determination was performed in the supernatant (12,000 g, 30′) of all formulations diluted in cell culture medium for the used *in vitro* concentrations with LAL chromogenic endotoxin quantitation kit. Results showed endotoxin values between 0.1 and 0.3 EU/mL.	Grabowski et al., 2016
PLGA NPs	75:25 Resomer^®^ RG756	170 nm −45 mV (200 nm in cell culture medium)	*In vitro* cytokine production (24 h incubation) (IL-8, IL-6, TNF-α, and MCP-1)	THP-1 cell culture (differentiated into macrophages)	0.1 or 1 mg/mL	0.1 mg/mL did not induce cytokine secretion 1 mg/mL induced IL-8 and TNF-α	–	Grabowski et al., 2015
PVA stabilized PLGA NPs	75:25 Resomer^®^ RG756 and PVA (87–89% hydrolyzed, 30–70 kDa)	230 nm −1 mV (210 nm in cell culture medium)	*In vitro* cytokine production (24 h incubation) (IL-8, IL-6, TNF-α, and MCP-1)	THP-1 cell culture (differentiated into macrophages)	0.1 or 1 mg/mL	0.1 mg/mL did not induce cytokine secretion 1 mg/mL induced IL-8	–	Grabowski et al., 2015
Chitosan stabilized PLGA NPs	75:25 Resomer^®^ RG756 and Protasan^®^ UP CL113,75–90% deacetylation, 50–150 kDa	230 nm + 40 mV (270 nm in cell culture medium)	*In vitro* cytokine production (24 h incubation) (IL-8, IL-6, TNF-α, and MCP-1)	THP-1 cell culture (differentiated into macrophages)	0.1 or 1 mg/mL	0.1 mg/mL and 1 mg/mL did not induce cytokine secretion^c^	–	Grabowski et al., 2015
Pluronic stabilized PLGA NPs	75:25 Resomer^®^ RG756 and Pluronic F68	230 nm −30 mV (315 nm in cell culture medium)	*In vitro* cytokine production (24 h incubation) (IL-8, IL-6, TNF-α and MCP-1)	THP-1 cell culture (differentiated into macrophages)	0.1 or 1 mg/mL	0.1 mg/mL did not induce cytokine secretion 1 mg/mL induced IL-6	–	Grabowski et al., 2015
PLGA NPs	PLGA lactic to glycolic acid 50:50 (intrinsic viscosity 0.60 g/dl) PLGA lactic to glycolic acid 65:35 (intrinsic viscosity 0.64 g/dl) PLGA lactic to glycolic acid 75:25 (intrinsic viscosity 0.72 g/dl) PLGA lactic to glycolic acid 85:15 (intrinsic viscosity 0.62 g/dl)	210 nm −14 mV 211 nm −8.70 mV 218 nm −12.7 mV 243 nm −12.7 mV	*In vitro* cytokine production (24 h incubation) (IL-6 and TNF-α)	RAW 264.7 cells	300 μg/mL	No induction of the IL-6 release 1.5- to 2-fold increase in TNF-α release	–	Singh and Ramarao, 2013
PLA NPs	DL-PLA (MW 10,000)	256 nm −17.1 mV	*In vitro* cytokine production (24 h incubation) (IL-6 and TNF-α)	RAW 264.7 cells	300 μg/mL	No induction of the IL-6 release 1.5- to 2-fold increase in TNF-α release	–	Singh and Ramarao, 2013
PCL NPs	PCL (intrinsic viscosity 1.07 g/dl)	268 nm −9.10 mV	*In vitro* cytokine production (24 h incubation) (IL-6 and TNF-α)	RAW 264.7 cells	300 μg/mL	No induction of the IL-6 release 1.5- to 2-fold increase in TNF-α release	–	Singh and Ramarao, 2013
poly(lactide-co-caprolactone) (PLCL) NPs	PLCL 25:75 (intrinsic viscosity 0.71 g/dl) PLCL 80:20 (intrinsic viscosity 0.77 g/dl)	261 nm −15.3 mV 261 nm −15.4 mV	*In vitro* cytokine production (24 h incubation) (IL-6 and TNF-α)	RAW 264.7 cells	300 μg/mL	No induction of the IL-6 release 1.5- to 2-fold increase in TNF-α release	–	Singh and Ramarao, 2013

a *Inferred results from the graphs. The authors do not show or discuss the comparison with non-treated cells*.

b *Only statistically significant increases were considered in the results*.

c *According to the authors, IL-6 levels were not statically different from the control but neither were LPS levels. Considering this, chitosan stabilized PLGA NPs induced IL-6 levels similar to LPS*.

For chitosan NPs, it is interesting to notice that one author referred chitosan NPs induced several cytokines in BMDCs (Koppolu and Zaharoff, 2013), while other did not (Han et al., 2016). Nevertheless, in both papers, no endotoxin contamination was assessed, no concentrations of NPs were given and the chitosan polymers and NPs characteristics were not the same. Furthermore, it must be considered that cytokine secretion highly depends on the cellular model under study. Indeed, Koppolu and Zaharoff, upon stimulation with chitosan NPs, reported the production of IL-1β in BMDCs and the absence of the same cytokine in RAW 264.7 (Koppolu and Zaharoff, 2013).

The fact that no endotoxin control was made in both papers can rise several questions, mainly in the results that suggest a positive stimulation of chitosan NPs. Chitosan has a cationic charge, resultant from the N-acetyl group removal during chitin deacetylation. This positive charge, mediates for instance the electrostatic interactions with cargo molecules, allowing high loading efficacies, but it also enables chitosan interactions with the negatively charged phosphate, pyrophosphate, and carboxylic groups of LPS (Davydova et al., 2000). Actually, chitosan has been used as a selective filtration membrane for endotoxin removal due to these extensive interactions (Machado et al., 2006; Lieder et al., 2013).

But not only chitosan should be evaluated regarding endotoxin contamination. For instance, Grabowski et al. have published two reports, comparing the inflammatory ability of different PLGA NPs based on the *in vitro* assessment of cytokines, such as IL-6, TNF-α, IL-8 and MCP-1 (Grabowski et al., 2015, 2016). The differences among PLGA NPs resulted from the inclusion of chitosan, PVA and P68 in order to obtain, positive, neutral and negatively charged particles. In one of the reports the authors do not evaluate or discuss the presence of endotoxin contamination in the formulations (Grabowski et al., 2015). Nonetheless, in the other report, using the same methods and polymers, the authors mentioned that all formulations presented 0.1 to 0.3 EU/mL of LPS depending on the concentration used (Grabowski et al., 2016). In both reports, this information was imperative, since the authors tested IL-8, IL-6 and TNF-α, cytokines whose production is induced by LPS (Agarwal et al., 1995; Grabowski et al., 2016). Therefore, despite their conclusions, as illustrated in Table 5 (Grabowski et al., 2015, 2016), and despite the authors attribute the observed effects to the nanoparticulate form of the formulations, the effect of LPS contamination might be interfering with the results. A simple control that could be adopted in this situation, was to use the LPS concentration the authors quantified in the formulations, incubate with the cell and assess the cytokine secretion. In these articles, the relationship between the 0.1–0.3 EU/ml of contamination and the 0.1–10 μg/mL of LPS as control was not given, and therefore, no further conclusions could be drawn regarding the effect of the LPS contamination in the formulations. Another relevant aspect to highlight, is the fact that nanoparticles, particularly polymeric nanoparticles interfere with most endotoxin quantification assays. This fact was denoted by the authors of these reports, who overcame the interference, by centrifuging the formulations and measuring the contamination in the supernatant (Grabowski et al., 2016). Unfortunately, due to what was discussed previously, the polymers, and particularly the positively charged, might adsorb the LPS through electrostatic interactions, which means the quantification on the supernatant can be underestimated. Overall, in this example, the conclusions about the mild inflammatory ability of PLGA and PLGA stabilized NPs should be extrapolated with caution, since the use of endotoxin free materials, or the presence of endotoxin inhibitor (i.e., polymycin B) might generate different results.

### Genotoxicity

Genotoxicity describes the capacity of the compounds to affect the DNA structure or the cellular apparatus and topoisomerases, modifying the genome fidelity (Słoczynska et al., 2014). Genotoxic effects are not always related with mutations but they can have serious implications for risks of cancer or chronic/heritable diseases (Słoczynska et al., 2014; Lorscheidt and Lamprecht, 2016; Dusinska et al., 2017).

NMs can cause damage to cell's DNA through direct and indirect interactions (Magdolenova et al., 2013; Lorscheidt and Lamprecht, 2016; Dusinska et al., 2017). In fact, upon cellular uptake, NMs might reach the nucleus and contact with cell genetic material, leading to physical or chemical alterations (Magdolenova et al., 2013; Lorscheidt and Lamprecht, 2016; Dusinska et al., 2017). Importantly, this direct interaction is limited by the particle size. Particles ranging between 8 and 10 nm of diameter may reach the nuclear compartment through nuclear pores, whether 15–60 nm particles will only access the nucleus during cellular division when the nuclear wall is disrupted (Barillet et al., 2010). However, indirect interactions have a greater significance for genotoxicity, since several biomolecules involved in normal gene function (i.e., DNA repair) and cell division (i.e., DNA transcription and replication) can interact with even larger NMs, altering its function and consequently leading to DNA injury or chromosome malformation (Lorscheidt and Lamprecht, 2016; Dusinska et al., 2017). For instance, oxidative stress is a key mechanism by which NMs can cause DNA injury (Dusinska et al., 2017). Therefore, data showing non-cytotoxic increase of ROS should imply genotoxicity studies to assess the degree of damage caused by the oxidative stress (Lorscheidt and Lamprecht, 2016).

Several assays are described in the literature for genotoxicity assessment and include *in vitro* and *in vivo* approaches. *In vitro* assays are commonly performed in cell lines, such as the mouse lymphoma L5178Y TK^+/−^ 3.7.2C cells, the TK6 human lymphoblastoid cells and rodent fibroblastic cell lines (CHL-IU, CHO and V79 cells) (Lorge et al., 2016). Regarding *in vivo* studies, the bacterial reverse mutation test (AMES test) is the most commonly used initial screening performed. Also, the *Allium cepa* model, allows for a simple and cost-effective assay where DNA damage is assessed after the roots of the plant grow in direct contact with the substance of interest (Bosio and Laughinghouse IV, 2012). Alternatively, other *in vivo* studies comprise the use of Zebrafish (*Danio rerio*) due to their molecular and physiological similarities with humans, therefore giving a high-throughput for genotoxicity (Chakravarthy et al., 2014). Rodents and other mammals are also widely used for genotoxicity assessment. In all these models, the comet assay, the micronucleus assay and the chromosome aberrations test are the most common used tests to evaluate nanoparticles toxicity (Magdolenova et al., 2013).

Importantly, some considerations have been published by OECD regarding the protocols to assess genotoxicity of NMs, namely the “2018 Report No. 85—Evaluation of *in vitro* methods for human hazard assessment applied in the OECD Testing Programme for the Safety of Manufactured Nanomaterials” and “2014 Report No. 43—Genotoxicity of Manufactured Nanomaterials: Report of the OECD expert meeting” (OECD, 2014, 2018a).

Data collected from the literature assessing genotoxicity of polymeric NMs is summarized in Table 6. Again, most of the data collected refers to chitosan and PLGA based NPs and should be carefully analyzed. First, we must recognize we are comparing NPs comprising a particular polymer (chitosan or PLGA) but whose chemical specifications can differ and whose composition and characteristics are very diverse. Also, comparisons should ideally be performed only when the same test is applied. In detail, chitosan/poly(methacrylic acid) NPs induced a concentration dependent genotoxic effect according to the cytogenetic test using human lymphocyte culture (De Lima et al., 2010). However, the same report reported no evidence for DNA alterations using the *Allium Cepa* assay (De Lima et al., 2010). In another study, Eudragit® S100/alginate enclosed chitosan calcium phosphate-loaded lactoferrin nanocapsules, was considered non-genotoxic based on the *Allium Cepa* and the comet assay in Vero cells (Leng et al., 2018). Overall, these two studies comprising nanoparticles with chitosan in their composition, presented a different conclusion for the NM genotoxicity, but if we compare only the same assay (*Allium Cepa* assay), the results were similar. Another interesting fact, is the heterogeneity of results that may be achieved with different cell lines. For instance, Platel et al. used three different cell lines, and three different PLGA NPs and evaluated genotoxicity using the comet assay and the micronucleus test (Platel et al., 2016). For bare PLGA NPs, no genotoxicity effects were verified in none of the 3 cell lines with both tests (Platel et al., 2016). On the other hand, CTAB stabilized PLGA NPs induced an increase in the number of micronuclei only in one of the cell lines (micronucleous test in HBE14o- cells) (Platel et al., 2016). These examples illustrate how an extrapolation based on one single genotoxicity assay (or cellular/animal model) can be misleading.

**Table 6:** Review of original articles assessing the genotoxicity of polymeric nanoparticles according to different testing methodologies.

**Nanomaterial**	**Polymer Characterization**	**Nanomaterial Characterization**	**Testing method**	**Model**	**Administration route (if applicable)**	**Dose/concentration range**	**Results**	**Observations**	**References**
Chitosan/poly(methacrylic acid) (CS/PMAA) NPs	Chitosan with 71.3 kDa and 94 % DD	60 nm 82 nm 111 nm	*Allium cepa* assay (24 h)	*Allium cepa* bulbs	–	1.8, 18, and 180 mg/L	No significant numerical or structural changes in DNA	Smaller particles were not toxic at higher concentrations, by opposition to larger size nanoparticles	De Lima et al., 2010
Chitosan/poly(methacrylic acid) (CS/PMAA) NPs	Chitosan with 71.3 kDa and 94 % DD	60 nm 82 nm 111 nm	Cytogenetic test	Human blood (lymphocyte culture)	–	1.8, 18, and 180 mg/L	The 82 and 111 nm NPs reduced mitotic index values at the highest concentration tested (180 mg/L)	Smaller particles were not toxic at higher concentrations, by opposition to larger size nanoparticles	De Lima et al., 2010
Eudragit^®^ S100/alginate-enclosed chitosan-calcium phosphate-loaded lactoferrin nanocapsules	na	240 nm −2.6 mV	*Allium cepa* assay (24 h)	*Allium cepa* bulbs	Roots immersed in formulations	125, 250, 500, and 1000 μg/mL	No genotoxicity	–	Leng et al., 2018
Eudragit^®^ S100/alginate-enclosed chitosan-calcium phosphate-loaded lactoferrin nanocapsules	na	240 nm −2.6 mV	Comet assay *(*24 h)	Vero cells	–	100 μg/mL	No genotoxicity	–	Leng et al., 2018
Poly-lactic-co-glycolic acid–polyethylene oxide (PLGA–PEO) NPs	na	143–180 nm −43 mV	Comet assay (24 h)	Human peripheral blood	–	3, 15, or 75 μg/cm^2^	No induction of SBs or oxidized DNA bases	–	Tulinska et al., 2015
Poly-lactic-co-glycolic acid–polyethylene oxide (PLGA–PEO) NPs	na	143–180 nm −43 mV	Micronucleous test (24 h)	Human peripheral blood	–	3, 15, or 75 μg/cm^2^	No increase in the number of micronucleated binucleated cells	–	Tulinska et al., 2015
PLGA NPs	Resomer^®^ RG503H, acid terminated, 50:50, Mw 24,000–38,000	80 nm −25 mV	Comet assay (3 h) and micronucleus test (3 + 40 h recovery time)	16HBE14o-, L5178Y and TK6 cells	–	50–500 μg/mL (16HBE14o-, L5178Y, and TK6 cells)	No primary DNA, no chromosomal damage and no increase in the number of micronulei on L5178Y and TK6 and 16HBE14o- cells	The L5178Y mouse lymphoma and TK6 human B-lymphoblastoid cells, are routinely used in *in vitro* regulatory genotoxic assays. The human bronchial epithelial cells 16HBE14o-, a cell line is suitable for toxicity studies of inhaled NPs as it is highly similar to the primary bronchial epithelium	Platel et al., 2016
PEG stabilized PLGA NPs	Resomer^®^ RG503H, acid terminated, 50:50, Mw 24,000–38,000	78 nm −1 mV	Comet assay (3 h) and Micronucleus test (3 + 40 h recovery time)	L5178Y and TK6 cells	–	50–500 μg/mL (L5178Y and TK6 cells)	No primary DNA, no chromosomal damage and no increase in the number of micronulei on L5178Y and TK6 cells	The L5178Y mouse lymphoma and TK6 human B-lymphoblastoid cells, are routinely used in *in vitro* regulatory genotoxic assays	Platel et al., 2016
hexadecyltrimethylammonium bromide (CTAB) stabilized PLGA NPs	Resomer^®^ RG503H, acid terminated, 50:50, Mw 24,000–38,000 and PEG 2000	82 nm +15 mV	Comet assay (3 h) and micronucleus test (3 + 40 h recovery time)	16HBE14o-, L5178Y and TK6 cells	–	25–100 μg/mL (L5178Y and TK6 cells) 25–100 μg/mL (16HBE14o- cells)	No primary DNA or chromosomal damage on L5178Y and TK6 cells; concentration-related increase in the number of micronuclei in 16HBE14o- cells	The L5178Y mouse lymphoma and TK6 human B-lymphoblastoid cells, are routinely used in *in vitro* regulatory genotoxic assays. The human bronchial epithelial cells 16HBE14o-, a cell line is suitable for toxicity studies of inhaled NPs as it is highly similar to the primary bronchial epithelium	Platel et al., 2016
Danorubicin loaded polyethylene glycol-poly L-lysine-poly lactic-co-glycolic acid (PEG-PLL-PLGA) NPs	na	229 nm −20 mV	*In vivo* exposure /bone marrow micronucleus assay	Kunming mice	Intravenous	1/2 LD_50_, 1/4 LD_50_, 1/8 LD_50_ per kg	No teratogenic or mutagenic effects		Guo et al., 2015
Poly(ε-caprolactone)-poly(ethylene glycol)-poly(ε-caprolactone) (PCEC) nanoparticles	PCEC copolymer with a molecular weight of 17,500 (1H NMR spectrum)	40 nm	Ames test (48 h)	*Salmonella typhimurium*	–	150–5,000 μg/mL	No mutagenicity to the *Salmonella typhimurium* strains TA97, TA98, TA100, TA102, and TA1535	–	Huang et al., 2010
Poly(ε-caprolactone)-poly(ethylene glycol)-poly(ε-caprolactone) (PCEC) nanoparticles	PCEC copolymer with a molecular weight of 17,500 (1H NMR spectrum)	40 nm	Chromosomal aberration test (6, 24, 48 h)	Chinese hamster lung (CHL) cells	–	150–5,000 μg/mL	No significant increases in the incidence of chromosomal aberrations	–	Huang et al., 2010
Poly(ε-caprolactone)-poly(ethylene glycol)-poly(ε-caprolactone) (PCEC) nanoparticles	PCEC copolymer with a molecular weight of 17,500 (1H NMR spectrum)	40 nm	Mouse micronucleus test (*in vivo* exposure, 1 or 2 administrations, 24 or 48 h)	ICR mice	Intraperitoneal	0, 0.4, 0.8, and 1.6 g/kg	No increase in micronuclei	–	Huang et al., 2010

### Toxicity on Reproduction

The extrapolation to human health of toxic effects on reproduction using *in vitro* and animal models presents several specific limitations, such as the differences in reproductive structures and endocrine functions or the duration of gestation or spermatogenesis period (Das et al., 2016). Also, alike other studies, the tested concentrations and doses are much higher than the clinically relevant doses in humans (Das et al., 2016). Nevertheless, the toxicity on reproduction is a valuable endpoint since it allows the prediction of health effects not only of individuals but also of the next generation (Dusinska et al., 2017).

As mentioned before, toxicity on reproduction might be evaluated using *in vitro* and *in vivo* studies. For instance, *in vitro* assays test the toxicity of nanoparticles in cells from reproductive organs (such as blastocysts and granulosa cells) or use *ex vivo* placentae or sperm from healthy donors (Ema et al., 2010; Sun et al., 2013; Brohi et al., 2017). In these examples, the authors expect to see direct toxicity of the NPs in reproductive system cells, or to evaluate the ability of the NPs to cross for instance the placental barrier (Ema et al., 2010; Brohi et al., 2017).

Regarding *in vivo* testing, the use of mice as a mammalian model provides analogous experimental conditions to humans. However, the investigation of early embryonic developmental effects occurring *in utero* are not easily detectable (Sun et al., 2013). Interestingly, the zebrafish model has been widely applied as a rapid and cost-effective whole animal model to assess reproductive toxicity (Hu et al., 2011). Characteristics like the small size, rapidity to reach sexual maturity, great number of eggs (200–300) and the possibility to examine every stage of embryonic development through its transparency, make zebrafish one of the most used animal models (Wang et al., 2016).

Results from toxicity on reproduction assays with polymeric NMs are summarized in Table 7. The results for chitosan NPs (blend and bare) are consistent between reports. In fact, it appears that chitosan based NPs induce embryonic malformations when directly in contact with embryos, or intravenously administered to animal models (Hu et al., 2011; Park et al., 2013; Choi et al., 2016; Wang et al., 2016; Yostawonkul et al., 2017). However, this effect is not verified in when PLGA NPs coated with chitosan are administered through the oral route in Sprague Dawley rats (Sharma et al., 2017). Though, this conclusion is only speculative. In order to have a proven conclusion, the oral route should be tested for toxicity on reproduction using the same NPs as were used for the intravenous administration and embryonic incubation experiments. Otherwise, we cannot be sure if the result is due to the administration route, or the NPs composition and characteristics. Nevertheless, other study using PLGA based NPs also tested toxicity on reproduction through the *in vitro* zebrafish embryonic model, and found no toxicity for those nanoparticles (Chen et al., 2017).

**Table 7:** Review of original articles assessing toxicity on reproduction induced by polymeric nanoparticles.

**Nanomaterial**	**Polymer characterization**	**Nanomaterial characterization**	**Testing method**	**Model**	**Administration route (if applicable)**	**Dose/concentration range**	**Results**	**Observations**	**References**
Chitosan NPs	na	100 nm	*In vivo* reproduction model/*in vitro* culture of embryos	ICR mice: Mouse pre-implantation embryos	–	10–200 μg/mL	Impaired blastocyst expansion and hatching Higher rates of resorption after embryo transfer Decreased implantation and increased embryonic death *in vivo*	Authors refer the use of different molecular-weight chitosan, derived from crab shell, without further distinctions	Park et al., 2013
Chitosan NPs	100 kDa and 85 % DD	200 nm	*In vitro* embryo model (72 h)	Zebrafish	–	5, 10, 20, and 40 μg/mL	Decrease in hatching rate (30 and 40 μg/mL) All embryos dies with 40 μg/mL Malformation with (5 μg/mL) Enhanced expression of ROS (5 μg/mL) Overexpression of HSP70 (5 μg/mL)	Dose dependent effect 200 nm nanoparticles showed higher toxicity than the 300 nm nanoparticles Results for ROS production were only presented for 5 μg/mL	Hu et al., 2011
Chitosan NPs	100 kDa and 95 % DD	85 nm	*In vitro* embryo model (5 days)	Zebrafish	–	100, 150, 200, 250, 300, 350, and 400 μg/mL	Dose-dependent effect in terms of malformation, mortality and hatching rates	The comparison between the toxicity of chitosan nanoparticles and chitosan powder suggested the nano assembly of chitosan was relatively more secure than normal chitosan particles	Wang et al., 2016
Chitosan NPs	na	100 nm	*In vitro* culture of embryos (24 h)	Mouse morula-stage embryos	–	100 μg/mL	Induce endoplasmic reticulum (ER) stress and double- and multi-membraned autophagic vesicles, that lead to cell death of blastocoels	–	Choi et al., 2016
Chitosan NPs	na	100 nm	*In vivo* reproduction model	ICR mice	Intravenous	500 μg/kg or 1,000 μg/kg b.w.^a^	Significant reduction in the number of developing follicles	–	Choi et al., 2016
Nanostructured lipid carrier (NLC)- oleoyl-quaternized-chitosan (CS)-coated	Chitosan (CS) (molecular weight 600 kDa)	147 nm + 44.9 mV	*In vitro* embryo model (incubation for 72 h)	Zebrafish	–	2.5, 5, 10, 20, and 40 μM	Embryonic survival was dose dependent exposure to 40 μM−100% embryo mortality Survivor embryos of the 5, 10, and 20 μM exposure presented some malformations (e.g., eye/head abnormalities, pericardial edema, and yolk sac edema)	Chitosan coating increased the toxicity of the NLC	Yostawonkul et al., 2017
Poly(lactic-*co*-glycolic acid) (PLGA)–polyethylene glycol (PEG)–folic acid (FA) NPs	PEG – MW 2kDa PLGA – MW 90 kDa (lactic to glycolic acid 50:50), carboxyl-terminated	131 nm −25 mV	*In vitro* embryo model (12 and 36 h) Zebrafish	Zebrafish	–	–	No serious malformation or death was observed at the embryo-development stage or for hatched zebrafish larva	–	Chen et al., 2017
Poly(lactic-*co*-glycolic acid) (PLGA) NPs	PEG – MW 2kDa PLGA – MW 90 kDa (lactic to glycolic acid 50:50), carboxyl-terminated	83 nm −27 mV	*In vitro* embryo model (12 and 36 h)	Zebrafish	–	–	No serious malformation or death was observed at the embryo-development stage or for hatched zebrafish larva	–	Chen et al., 2017
Polyphenolic bio-enhancers with oleanolic acid in chitosan coated PLGA NPs (CH-OA-B-PLGA NPs)	Chitosan (molecular weight 150 kDa, deacetylation degree 85%), Poly (lactide-coglycolide) (PLGA) 50:50, mw 40–75 kDa	342 nm + 34 mV	*In vivo* exposure (21 days)	Sprague Dawley rats	Oral	100 mg/kg b.w. of OA	Normal mating Major increase in the weight Higher number of pups at parturition No sign of abnormality or deformation on pups	100 mg/kg is the double of the OA effective dose	Sharma et al., 2017
Polyphenolic bio-enhancers with oleanolic acid in PLGA NPs (OA-B-PLGA NPs)	Poly (lactide-coglycolide) (PLGA) 50:50, mw 40–75 kDa	221 nm −19 mV	*In vivo* exposure (21 days)	Sprague Dawley rats	Oral	100 mg/kg b.w. of OA	Authors do not present or discuss the result	100 mg/kg is the double of the OA effective dose	Sharma et al., 2017

a *b.w., body weight*.

### Hemocompatibility

Hemocompatibility is frequently assessed as an endpoint of biocompatibility for chemicals and particularly NMs. In fact, blood is the first target when considering intravenous injections of NMs, but it is also a surrogate target model for other routes of exposure, since its high complexity allows for an approximation the overall body response (Tulinska et al., 2015).

In particular, hemolysis which is associated to red blood cells damage is believed to have a good correlation with toxicity, since the *in vitro* hemolytic assays show results that greatly relate with *in vivo* toxicity studies (Dobrovolskaia and McNeil, 2013).

In 2008, Dobrovolskaia et al. published a report describing the validation of an *in vitro* assay for the analysis of nanoparticle hemolytic properties and main interferences (Dobrovolskaia et al., 2008). In 2013, ASTM International standards organization published the Standard Test Method for Analysis of Hemolytic Properties of Nanoparticles and defined a material as hemolytic if the hemolysis values are above 5% and as moderately hemolytic if they are between 2 and 5% (ASTM International, 2013; Dobrovolskaia and McNeil, 2013). Therefore, the existence of this protocol contributes to the use of standardized procedures among research groups, allowing comparisons and extrapolations of results.

From Table 8 we can acknowledge several authors reporting the hemolytic activity of diverse polymeric NMs. An important remark is the fact that a number of papers describe the hemolytic activity of drug loaded formulations and compare it to the free drug, but not with the unloaded nanocarrier (Essa et al., 2012; Gupta et al., 2012; Altmeyer et al., 2016; Radwan et al., 2017a). These results generally demonstrate a lower hemolysis rate of the drug loaded polymeric NM in comparison to the free drug, but still a significant hemolysis (>5%) (Essa et al., 2012; Gupta et al., 2012; Radwan et al., 2017a). In these situations, no conclusion regarding the hemolytic activity of the polymeric NM itself can be drawn. On the other hand, some other authors, test the unloaded nanoparticles but make no disclosure of their concentration (Altmeyer et al., 2016; Moraes Moreira Carraro et al., 2017).

**Table 8:** Review of original articles assessing hemolysis induced by polymeric nanoparticles.

**Nanomaterial**	**Polymer characterization**	**Nanomaterial characterization**	**Testing method**	**Model**	**Dose/concentration range**	**Results**	**Observations**	**References**
Chitosan NPs	270 kDa	367 nm +5 mV	Erythrocyte incubation (2 h)	Human blood	2000 μg/mL	Chitosan NPs were slightly hemolytic (~7%)	–	Shelma and Sharma, 2011
Chitosan NPs	Low molecular weight chitosan ≥75% DD	180 nm + 48 mV (acetic acid) 150 nm +39 mV (lactic acid) 140–160 nm +(20–25) mV (saline)	Whole blood incubation (3 h)	Human blood	50 μg/mL	NPs prepared in acetic acid medium showed high % hemolysis compared to those prepared in lactic acid medium, whereas the saline-dispersed NPs were found to be hemocompatible	The authors also tested the molecular chitosan and was hemocompatible	Nadesh et al., 2013
Chitosan NPs	Low molecular weight chitosan (85% DD)	≤ 100 nm +40 mV	Erythrocyte incubation (2 h)	Human blood	50–300 μg/mL	No significant hemolysis	Bulk chitosan was tested at the same concentrations.	Sarangapani et al., 2018
Chitosan NPs	50 kDa and 85% DD	~300 nm +35 mV	Erythrocyte incubation (2, 4 h)	Wistar rat	2.5 and 3.75 mg/mL	Low hemolysis rates		Kumar et al., 2017
Oleoyl-carboxymethyl-chitosan (OCMCS) nanoparticles	170 kDa chitosan, 92.56% DD modified with chloroactic acid and oleoyl chloride	171 nm +19 mV	Erythrocyte incubation (30, 60 min)	Carp blood	1 and 2 mg/mL	No hemolysis		Liu et al., 2013
PLA NPs	Poly(D,L-lactide) (PDLLA) 101782 g/mol and 0.68 dL/g	188 nm −24 mV (water) 109 nm −7 mV (water)	Whole blood incubation (3 h)	Human blood	38, 50, 200, 250 μg/mL	No hemolysis		Da Silva et al., 2019
PLA NPs	Poly(D,L-lactide) (PDLLA) 101782 g/mol and 0.68 dL/g	188 nm −24 mV (water) 109 nm −7 mV (water)	Whole blood incubation (3 h)	Human blood	75, 100, 300, 400 μg/mL	No hemolysis		Da Silva et al., 2019
Amphotericin loaded PEG-PLGA NPs	Copolymer produced with 6000 Da PLGA (lactic to glycolic acid molar ratio of 1:1) and 15% PEG	25 nm	Erythrocyte incubation (8 and 24 h)	Sprague Dawley Rat blood	Equivalent to 20, 50, and 100 μg/mL of amphotericin	Low hemolysis rate (<15%) Concentration dependent	Reduced hemolysis when compared to amphotericin commercial formulation (same dose)	Radwan et al., 2017a
Amphotericin loaded PEG-PLGA NPs	PLGA lactic to glycolic acid 50:50 with 40–75 KDa and PEG with 10 KDa	170 nm	Erythrocyte incubation (1 h)	Human blood	Equivalent to 25 μg/mL of amphotericin	Nanoparticles reduced the hemolytic activity of amphotericin in more than 95% Blank nanoparticles induced negligible hemolysis (unknown concentration)		Moraes Moreira Carraro et al., 2017
Amphotericin loaded PLGA NPs	PLGA lactic to glycolic acid 50:50 with 40–75 KDa	190 nm	Erythrocyte incubation (1 h)	Human blood	Equivalent to 25 μg/mL of amphotericin	Nanoparticles reduced the hemolytic activity of amphotericin in more than 95% Blank nanoparticles induced negligible hemolysis (unknown concentration)		Moraes Moreira Carraro et al., 2017
Casein stabilized PLGA NPs	PLGA lactic to glycolic acid 75:25, 5,000 kDa PEI: 25 kDa	165 nm −21 mV	Diluted whole blood incubation (3 h)	Human blood	0.01–10 mg/mL	No hemolysis		Pillai et al., 2015
PVA stabilized PLGA NPs	PLGA lactic to glycolic acid 75:25, 5,000 kDa PEI: 25 kDa	159 nm −0.14 mV	Diluted whole blood incubation (3 h)	Human blood	0.01–10 mg/mL	No hemolysis		Pillai et al., 2015
PEI stabilized PLGA NPs	PLGA lactic to glycolic acid 75:25, 5,000 kDa PEI: 25 kDa	158 nm +30 mV	Diluted whole blood incubation (3 h)	Human blood	0.01–10 mg/mL	7% hemolysis at the highest concentration tested (10 mg/ml)		Pillai et al., 2015
Acyclovir loaded Galactosylated (Gal)-PLGA NPs	na	173 nm −20 mV	Erythrocyte incubation (3 h)	na	0.1 mM of acyclovir	3.3% hemolysis	Free acyclovir in the same concentration induced 16.7% hemolysis	Gupta et al., 2012
Acyclovir loaded PLGA NPs	na	198 nm −8.5 mV	Erythrocyte incubation (3 h)	na	0.1 mM of acyclovir	9.8% hemolysis	Free acyclovir in the same concentration induced 16.7% hemolysis	Gupta et al., 2012
Poly(lactic-*co*-glycolic acid) (PLGA)–polyethylene glycol (PEG)–folic acid (FA) NPs	PEG – MW 2kDa PLGA – MW 90 kDa (lactic to glycolic acid 50:50), carboxyl-terminated	131 nm −25 mV	Diluted whole blood incubation (1 h)	New Zeeland Rabbit blood	0.033, 0.05, and 0.1 mg/mL	No significant hemolysis (<4%)		Chen et al., 2017
Poly(lactic-*co*-glycolic acid) (PLGA) NPs	PEG – MW 2 kDa PLGA – MW 90 kDa (lactic to glycolic acid 50:50), carboxyl-terminated	83 nm −27 mV	Diluted whole blood incubation (1 h)	New Zeeland Rabbit blood	0.033, 0.05, and 0.1 mg/mL	No significant hemolysis (<4%)		Chen et al., 2017
Danorubicin loaded polyethylene glycol-poly L-lysine-poly lactic-co-glycolic acid (PEG-PLL-PLGA) NPs	na	229 nm −20 mV	Erythrocyte incubation (15 min−3 h)	New Zeeland Rabibit blood	50 mg/mL (unloaded)	No hemolysis		Guo et al., 2015
Tamoxifen loaded PLA NPs	85–160 kDa PLA	155 nm −21.7 mV	Erythrocyte incubation (4, 12, 24,48, 72, 96 h)	Human blood	4.4 or 1.1 μM of tamoxifen	Negligible hemolysis at both concentrations and all incubations times	No results presented for blank NPs but is stated they cause no cellular damage to erythrocytes	Altmeyer et al., 2016
Itraconazole loaded PLA NPs	PLA (molecular weight: 56,000	284 nm ~0 mV	Erythrocyte incubation (3 h)	Wistar rat blood	5–20 μg/mL of ITZ i.e., 53–212 μg/mL of NPs	Significant hemolysis (>5%), concentration dependent	Reduced hemolysis when compared to free itraconazol (same dose). Hemolysis is suggested to be caused by the drug release during incubation	Essa et al., 2012
Itraconazole loaded PEG-PLA NPs	PEG7%-g-PLA, molecular weight: 8,300	197 nm ~0 mV	Erythrocyte incubation (3 h)	Wistar rat blood	5–20 μg/mL of ITZ i.e., 35–142 μg/mL of NPs	Significant hemolysis (>5%), concentration dependent	Reduced hemolysis when compared to free itraconazol (same dose). Hemolysis is suggested to be caused by the drug release during incubation	Essa et al., 2012
Itraconazole loaded PEG-PLA NPs	[PLA–PEG–PLA]n, molecular weight: 3,900	185 nm ~0 mV	Erythrocyte incubation (3 h)	Wistar rat blood	5–20 μg/mL of ITZ i.e., 40–159 μg/mL of NPs	Significant hemolysis (>5%), concentration dependent	Reduced hemolysis when compared to free itraconazol (same dose). Hemolysis is suggested to be caused by the drug release during incubation	Essa et al., 2012
Paclitaxel loaded monomethoxypoly (ethylene glycol)-b-poly(lactic acid) (mPEG-PLA) polymeric micelles	mPEG-PLA copolymer (40/60) with a number average molecular weight of 4488.4 and mPEG-PLA copolymer (50/50)	(40/60): 37 nm After incubation with BSA: 40 nm (50/50): 44 nm After ncubation with BSA: 71 nm	Erythrocyte incubation (1 h)	New Zeeland rabbit blood	2–10%	Minimal hemolysis (<6%)	The toxicity of paclitaxel loaded mPEG-PLA (40/60) polymeric micelles was significantly lower than those of mPEG-PLA (50/50)	Li et al., 2014

Nevertheless, polymeric NMs appear to present good hemocompability profile, as in most tested cases, hemolysis is a concentration dependent phenomenon, reaching significant values only for high NM concentrations. Also, the encapsulation of hemolytic drugs in polymeric NMs decreases their hemolytic activity.

## Discussion

Most information available on nanotoxicity is related to inorganic NMs, such as zinc oxide NPs, nanoscale silver clusters, and titanium dioxide NPs or carbon nanotubes (Yuan et al., 2015). Information related to polymeric NMs toxicity that could be correlated with their effects on human health is still scarce and poorly harmonized.

The majority of reports on polymeric NMs are focused in optimizing the nanocarrier features, such as size, physical stability and drug loading efficacy, and in performing preliminary cytocompatibility testing (mainly through MTT and LDH assays) and proving effectiveness of the drug loaded formulation, using the most diverse cell lines (Lorscheidt and Lamprecht, 2016). Toxicological studies exploring the biological effects of the polymeric NMs, particularly regarding immune system interaction are often disregarded. Though, as suggested by the safe-by-design concept, the toxicity study of NMs should be the starting point for the formulation development.

After our research on original peer reviewed articles, we selected the following endpoints to analyze that are crucial to understand the toxicity of nanobiomaterials for drug delivery: acute toxicity, repeated-dose toxicity, inflammation, oxidative stress, genotoxicity (including carcinogenicity and mutagenicity) toxicity on reproduction, and hemolysis. Importantly, one of the first conclusions to retain is that among different research groups, the methodologies, the animal or cellular model, the dose or concentration, the assay duration and notably, the polymeric NM properties, are not the same, making it difficult to compare and establish trends. This issue derives in part from the absence of regulatory binding and standardized methodologies and guidelines which hardens the comparison of safety/toxicity assessments in different reports (Dhawan and Sharma, 2010), and ultimately, makes it difficult to extrapolate safety profiles for human health. A similar conclusion was achieved by Park and coworkers, who discussed the status of *in vitro* toxicity studies for wide-ranging NMs, particularly cytotoxicity, oxidative stress, inflammation and genotoxicity and established that important limitations were preventing their use for human health risk assessment (Park et al., 2009).

Among the different polymeric NMs available, the most studied and reported are chitosan and PLGA nanoparticles. “Chitosan nanoparticles” and “PLGA nanoparticles” are general terms used for an endless number of different nanoparticles comprising multiple polymeric combinations, cross-links and surfactants, and therefore, displaying diverse physical and chemical properties as illustrated by the first 3 columns of Tables 3–8. As expected, these variables, together with the great diversity of protocols employed by different authors for the same assays, generates ambiguous results that prevent the establishment of trends between the nanocarriers characteristics and the expected toxicological endpoints.

An adequate characterization of the polymeric NMs is crucial for a comprehensive interpretation of the results but also to allow a comparison between different NMs. In 2018, in the context of EU FP-7 GUIDEnano project, it was published the development of a systematic method to assess similarity between NMs that would allow the extrapolation of results for human hazard evaluation purpose (Park et al., 2018). In that methodology they defined the following parameters for assessing similarities between NMs: chemical composition, crystalline form, impurities, primary size distribution, aggregate/agglomerate size distribution, density, and shape. Importantly, those parameters should be tested and compared in relevant media accordingly to the exposure route or toxicity test. However, in the process of developing such methodology, the authors identified several challenges that prevented the establishment of thresholds for establishing similarity. They suggest that the awareness of researchers for the relevance of characterizing NMs when performing hazard assessments is increasing which can lead to the establishment of the thresholds in the future, facilitating the extrapolation of hazard endpoints between similar NMs. Indeed, among the different research articles analyzed, the lack of broad characterization is frequent, sometimes even ignoring important parameters, such as the polymer molecular weight or the nanoparticle size.

Another aspect that should be taken into consideration when characterizing the polymeric NMs to study their biological effects is the endotoxin contamination. In fact, when discussing for instance cytokine stimulation or oxidative stress, endotoxin contamination should not be neglected. Nevertheless, endotoxin quantification (or its acknowledgment) on chitosan and other polymeric NMs is still scarce, which compromises some of the results found in the literature regarding their bioactivity and toxicity. In addition, despite testing the presence of endotoxins is a common procedure in laboratory and several commercial tests are available, they need to be validated for use with NMs, since most are based on optical assays and may be affected by the optical density of NPs (Dobrovolskaia et al., 2010).

Not only endotoxin detection assays are susceptible of interference from NMs and consequently misinterpretation of the results. Therefore, one way of trying to overcome this problem is to use different assays to evaluate the same endpoint. Additionally, experiment controls, such as the incubation of probes (without biological matrixes) and positive controls with NMs, can reveal whether these NMs might be generating false positive or negative results.

The obstacles identified in this review prevent the identification of toxicity trends and the generation of a useful database where we can rely for the Safe-by-Design. Only by performing *in vitro* and *in vivo* harmonized toxicity studies using unloaded polymeric NMs, extensively characterized regarding their intrinsic and extrinsic properties and by performing all necessary controls it is possible to generate such database. At the present time, taking everything into account, the human health risk assessment of polymeric NMs is still dependent on a case-by-case evaluation, and it should comprise the evaluation of parameters, such as the route of administration and dose, among others, to define the required tests for the hazard assessment (i.e., type of *in vitro* and *in vivo* studies).

## Author Contributions

SJ gathered the information, analyzed, and wrote the first draft of the manuscript. SJ, MS, CS, GB, PW, and OB defined the subjects for discussion. All authors contributed to manuscript revision, read, and approved the submitted version.

### Conflict of Interest

The authors declare that the research was conducted in the absence of any commercial or financial relationships that could be construed as a potential conflict of interest.

## Footnotes

^1^OECD. Available online at: http://www.oecd.org/science/nanosafety/publications-series-safety-manufactured-nanomaterials.htm (accessed June 15, 2018).
